# Amorfrutin B Compromises Hypoxia/Ischemia-induced Activation of Human Microglia in a PPARγ-dependent Manner: Effects on Inflammation, Proliferation Potential, and Mitochondrial Status

**DOI:** 10.1007/s11481-024-10135-9

**Published:** 2024-07-01

**Authors:** Karolina Przepiórska-Drońska, Agnieszka Wnuk, Bernadeta Angelika Pietrzak-Wawrzyńska, Andrzej Łach, Weronika Biernat, Anna Katarzyna Wójtowicz, Małgorzata Kajta

**Affiliations:** 1 Maj Institute of Pharmacology, Polish Academy of Sciences, Department of Pharmacology, Laboratory of Neuropharmacology and Epigenetics, Smetna Street 12, 31–343 Krakow, Poland; 2 Faculty of Animal Sciences, Department of Nutrition, Animal Biotechnology and Fisheries, University of Agriculture, Adama Mickiewicza 24/28, 30-059 Krakow, Poland

**Keywords:** HMC3, Microglia, Inflammation, Selective PPARγ modulator, Perinatal asphyxia, Stroke

## Abstract

Amorfrutin B is a selective PPARγ modulator that we demonstrated to be a promising neuroprotective compound in cellular models of stroke and perinatal asphyxia. Although neuronal mechanisms of amorfrutin B-evoked neuroprotection have been identified, none of them reflects the actions of the compound on microglia, which play a pivotal role in brain response to hypoxia/ischemia. Here, we provide evidence for amorfrutin B-induced effects on human microglia subjected to hypoxia/ischemia; the compound counteracts inflammation, and influences mitochondrial status and proliferation potential in a PPARγ-dependent manner. Post-treatment with amorfrutin B decreased the IBA1 fluorescence intensity, reduced caspase-1 activity, and downregulated *IL1B/*IL-1β and *TNFA* but not *IL10/*IL-10 expression*,* which was upregulated. Amorfrutin B also stimulated PPARγ signaling, as evidenced by increased mRNA and/or protein levels of PPARγ and PGC1α. In addition, amorfrutin B reversed the hypoxia/ischemia-evoked effects on mitochondria-related parameters, such as mitochondrial membrane potential, *BCL2*/BCL2 expression and metabolic activity, which were correlated with diminished proliferation potential of microglia. Interestingly, the inhibitory effect of amorfrutin B on the proliferation potential and mitochondrial function of microglia is opposite to the stimulatory effect of amorfrutin B on mouse neuronal survival, as evidenced by increased neuronal viability and reduced neurodegeneration. In summary, this study showed for the first time that amorfrutin B compromises hypoxia/ischemia-induced activation of human microglia in a PPARγ-dependent manner, which involves inhibiting inflammation, normalizing mitochondrial status, and controlling proliferation potential. These data extend the protective potential of amorfrutin B in the pharmacotherapy of hypoxic/ischemic brain injury, targeting not only neurons but also activated microglia.

**Graphical Abstract:**

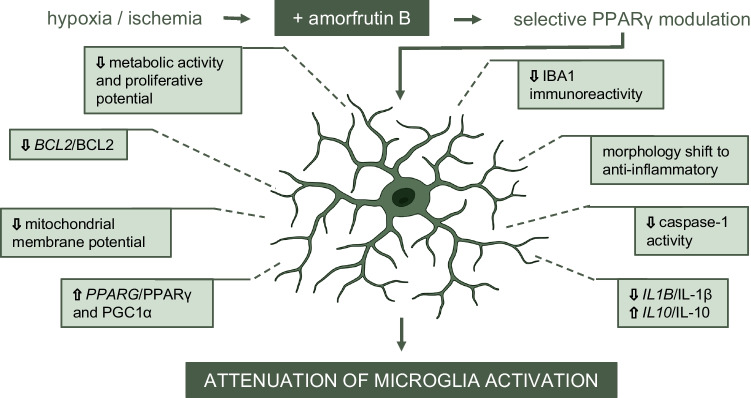

**Supplementary Information:**

The online version contains supplementary material available at 10.1007/s11481-024-10135-9.

## Introduction

Perinatal asphyxia and ischemic stroke are the leading causes of newborn and adult mortality, and they are serious challenges in current medicine. Hypothermia is the primary therapeutic strategy used for perinatal asphyxia; however, approximately 40% of neonates receiving this treatment develop adverse effects, e.g., hypotension and hemodynamic instability of the cardiopulmonary system (Popescu et al. 2020; Geisinger et al. 2024). In turn, the available treatment for stroke is recombinant tissue plasminogen activator (rtPA), but only 1–5% of patients with ischemic stroke presenting within 4.5 h of symptoms receive this medicine (Jilani and Siddiqui 2023). The unpredictable onset and course of perinatal asphyxia and stroke, as well as the lack of effective therapy without adverse side effects, make identifying an efficient treatment for these brain pathologies a real challenge. Because inflammatory processes determine both the first and later stages of perinatal asphyxia and stroke pathology (Danladi and Sabir 2021), it is widely accepted that modulating microglial function in the brain may inhibit neuroinflammation and, in this way, promote recovery. Interestingly, peroxisome proliferator-activated receptor gamma (PPARγ), a transcription factor that is the molecular target of antidiabetic thiazolidinedione (TZD), is a master regulator of the shift in microglial polarization from activated to quiescent. Full PPARγ agonists, such as pioglitazone, reduce the immunoreactivity of microglia during pathological conditions, preventing neuronal loss (Machado et al. 2019), however, clinical trials have indicated severe side effects (hepatotoxicity or cardiotoxicity) of TZD, which has led to its partial withdrawal from the pharmaceutical market (Zhong et al. 2018; Tang et al. 2018; El-Din et al. 2021).

Current efforts aim to optimize the structure of PPARγ ligands and deprive them of dangerous side effects. An interesting candidate with these properties is plant-derived amorfrutin B, a novel selective PPARγ modulator (SPPARγM), which activates the receptor in distinct ways depending on the cellular context and different receptor conformations (Grygiel-Górniak 2014). To date, amorfrutin B has been shown to elicit positive outcomes in mice with insulin resistance, liver steatosis and dyslipidemia (Weidner et al. 2013). In the cited study, male mice were fed with high-fat diet to induce obesity and insulin resistance. Next, the effects of amorfrutin B incorporated in the food were assessed with the use of intraperitoneal insulin sensitivity test and metabolic variable measurements. Amorfrutin B improved insulin sensitivity, glucose tolerance and blood lipid variables, did not induce weight gain and appeared a liver-protecting compound. In addition, in vivo studies have indicated that these properties are not accompanied by side effects typical of TZDs, such as osteoblastogenesis or fluid retention (Lavecchia and Di Giovanni 2015). Our team was the first to demonstrate that amorfrutin B protects primary mouse neurons against hypoxia/ischemia-induced cell damage, even when applied 6 h after injury. We showed that this PPARγ-specific neuroprotection relies on epigenetic modifications, as well as inhibition of apoptosis, autophagy and oxidative stress (Wnuk et al. 2021a, Wnuk, Przepiórska et al. 2021; Przepiórska et al. 2023). The strong effectiveness of amorfrutin B in protecting cells against hypoxia/ischemia suggests that this compound has the potential to adjust to clinical standards, however, it is unknown whether its possible use may be applicable to modulating microglial immunoreactivity in pathological conditions caused by hypoxia and ischemia.

Recent studies have indicated that acute ischemic stroke contributes to a mean loss of 2.03 million neurons or even 14.8 billion synapses per minute in the human brain (Desai et al. 2019). Hypoxic/ischemic events induce brain damage, primarily causing death of cerebral neurons localized in ischemic core. In the surrounding penumbra, some neuronal cells survive becoming the targets for therapy. Initially, brain neurons die due to hypoxia/ischemia-induced excitotoxicity, oxidative stress and necrosis, but later apoptosis and neuroinflammation are prerequisites for cell death (Muzio et al. 2021). Oxidative stress and inflammatory processes may contribute to the severity of brain damage during reperfusion, persisting even for several days after a hypoxic/ischemic episode (Mollet et al. 2022). Importantly, neuroinflammation can play a beneficial role in maintaining brain homeostasis, but when excessively activated it can play a pathological role, damaging brain cells and destroying neurological function. The pathogenesis of various neurological diseases, such as cerebrovascular disease, Alzheimer’s disease, Parkinson’s disease, and multiple sclerosis, has been closely linked to neuroinflammation (Tian et al. 2022).

Microglia are resident immune cells that represent an equal 10% of the adult brain cell population and participate in inflammatory reactions, acting as the first line of defense during neurodegenerative disorders such as stroke (Ochocka and Kaminska 2021; Li et al. 2020). After ischemia, in the ischemic core, cells with damaged cell membrane e.g., necrotic neurons, generate signals (glutamate, ATP, cytokines or HSP60) stimulating production of inflammatory cytokines and chemokines, and inducing morphological and phenotypic shifts in microglia (Yenari et al. 2010). The dynamics of microglial response during ischemic stroke may be divided into three stages: the acute phase (hours), subacute phase (days to weeks) and chronic phase (weeks to years), while microglia become activated and start proliferating reaching peak values 1–4 days after stroke (DeLong et al. 2022; Fan et al. 2023). A rich variety of ischemic stimuli lead to generation of distinct microglia phenotypes including those exhibiting proinflammatory or dysfunctional traits (e.g., foam cells) and others showing pro-repair features (Planas 2024). Considering that the microglial immune response takes hours to days, microglia represents a target for therapeutic intervention with an accessible time window (Yenari et al. 2010).

In addition to the pathological role of microglia in neurodegenerative diseases including stroke, beneficial roles of microglia have become apparent. These include an ability to regulate brain homeostasis and orchestrate postnatal connectivity in healthy brain (Rayasam et al. 2021). Microglia are commonly believed to play dual roles by presenting an M1-like (proinflammatory) or M2-like (anti-inflammatory) phenotype and exerting detrimental or beneficial effects during different stages of hypoxia/ischemia. The initial inflammatory response after ischemic brain injury involves microglial activation and the transition to classic M1-like phenotype, which is manifested by amoeboid cell morphology, caspase-1 activation, increased IBA1 level and the secretion of proinflammatory cytokines, including TNF-α and IL-1β (Taylor and Sansing 2013; Li et al. 2019; Xu et al. 2020). The binary concept of "M1" and "M2" phenotypes and microglial polarization paradigms during inflammatory activation were widely accepted well into twenty-first century, leading to a surge in articles phenotyping microglia into deleterious “M1” or beneficial “M2” in the 2010s. With the identification of a broad repertoire of microglial states and functions dependent on developmental stage, plasticity, aging, and disease, the binary concept has become incoherent. Currently, a dynamic concept of microglial states that takes into account microglial function is postulated (Paolicelli et al. 2022). To account for the new concept, expressions “M1-like” and “M2-like” were used instead of “M1” and “M2” in this study.

The present study aimed to determine whether amorfrutin B is an effective modulator of the immune response in microglia activated by hypoxia/ischemia. For that reason, we used the human microglial HMC3 cell line, which is a suitable material for biochemical and molecular analyses and shows expression patterns of cytokines and chemokines similar to those of human primary microglia (Baek et al. 2021). We hypothesize that targeting PPARγ/PGC1α signaling with amorfrutin B diminishes hypoxia/ischemia-induced microglial activation and contributes to the inhibition of inflammation, a shift in microglial morphology to a quiescent phenotype and mitochondria-dependent control of proliferation potential. We decided to complement our studies on microglia by performing an experiment on primary cortical neurons indicating that, in the future therapy, amorfrutin B should protect neurons and modulate microglial activity. In our opinion, the unique ability of amorfrutin B to regulate the microglial response will support its neuroprotective potential for the future treatment of stroke and perinatal asphyxia.

## Materials and Methods

### Materials

A hypoxia modular incubator chamber (MIC-101) was acquired from Billups-Rothenberg, Inc. (San Diego, CA, USA). We used the following CO_2_ incubators; New Brunswick Galaxy 170 R Stainless Steel CO_2_ Incubator and New Brunswick Innova CO-170 CO_2_ Incubator, both acquired from New Brunswick Scientific (Edison, New Jersey, USA). Phosphate-buffered saline (PBS – MS01P01003) was purchased from Biowest (Nuaillé, France). Fluoro-Jade C Dry Powder (TR-160-FJC) was obtained from Biosensis (Thebarton, Australia). JC-1 (#30001) was obtained from Biotium, Inc. (Hayward, CA, USA). B27 (10889–038), Neurobasal medium (with glucose: 12348–017; without glucose: 12015621), and DMEM (with glucose: 31053–028; without glucose: A1443001) were obtained from Gibco (Grand Island, NY, USA). The oxygen analyser (GOX100 Cat. No. 600437) was from Greisinger (Regenstauf, Germany). The Bradford assay (5000006) was obtained from Bio-Rad Laboratories (Munchen, Germany). ELISA kits for IL-1β (E0143Hu), IL-10 (E0102Hu), PPARγ (E1511Hu), PGC1α (E3509Hu), and BCL2 (BPE040) were purchased from Bioassay Technology Laboratory (Shanghai, China). The culture plates (6-well: 92006, 24-well: 92024, 96-wells: 92096) were obtained from TPP Techno Plastic Products AG (Trasadingen, Switzerland), and 75 cm^2^ U-shaped canted neck cell culture flasks (353136) with a vent cap was obtained from Corning (New York, USA). HMC3 cells (CRL-3304™, Lot number: 70046457), EMEM (30–2003), FBS (30–2020), and trypsin/EDTA (30–2101) were purchased from American Type Culture Collection (ATCC—Virginia, USA). Fast Probe qPCR Master Mix (E0422-03) was from EurX (Gdansk, Poland). The Cytotoxicity Detection Kit (11644793001) was purchased from Roche Diagnostics GmbH (Mannheim, Germany). The primary anti-IBA1 (sc-32725) antibody was purchased from Santa Cruz Biotechnology, Inc. (Santa Cruz, CA, USA). Amorfrutin B (SMB00532), GW9662 (M6191), *L*-glutamine (G-8540), FBS (F7524), DMSO (D8418), RIPA (89,901) buffer, protease inhibitor cocktail for mammalian tissues (P8340), penicillin–streptomycin antibiotics (P4333), caspase-1 substrate (SCP0070), MTT (M5655), BrdU cell proliferation assay (QIA58), calcein AM (C1359), and poly-*L*-ornithine hydrobromide (P4538) were obtained from Sigma‒Aldrich (St. Louis, MO, USA). The RNeasy Mini Kit (74106) was obtained from Qiagen (Hilden, Germany). Alexa Fluor 647 IgG (A21235), a high-capacity cDNA reverse transcription kit (436881), alamarBlue™ Cell Viability Reagent (DAL1101), Hoechst 33342 (62249), and TaqMan probes (4331182) for specific genes encoding *ACTB*, *GAPDH*, *HPRT*, *IL1B*, *IL10*, *TNFA*, *PPARG*, *PGC1A,* and *BCL2* were obtained from Thermo Fisher Scientific (Waltham, MA, USA).

### Human Microglial HMC3 Cell Line

**Fig. 1:**
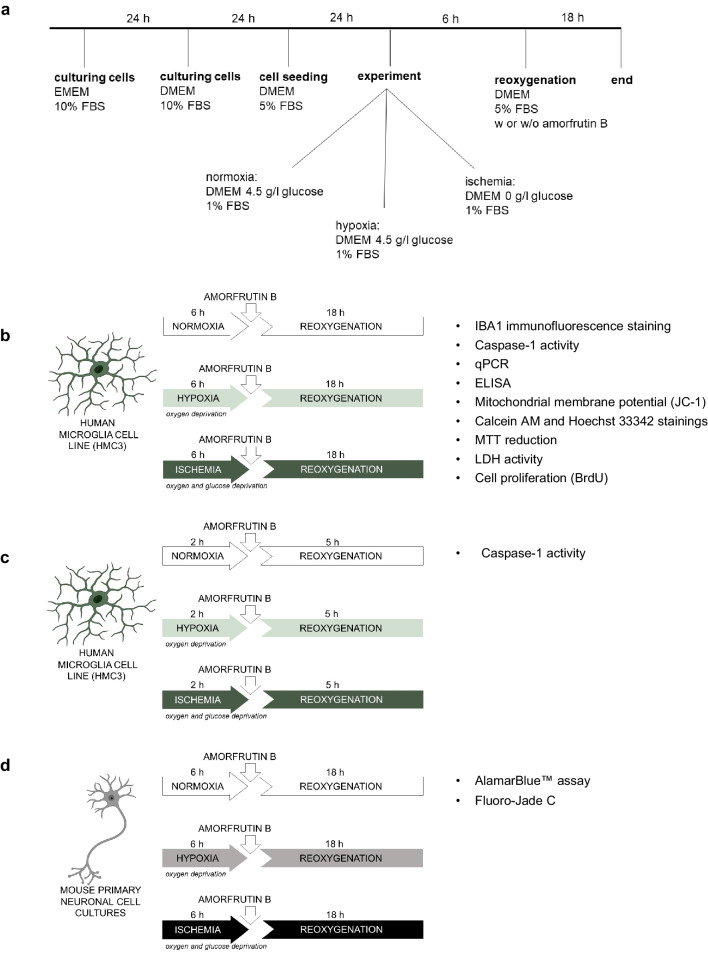
The timeline showing experimental conditions from culturing to the end of the experiment (**a**). Experimental models of hypoxia/ischemia and amorfrutin B post-treatment in human microglia cell line HMC3 (**b** and **c**) and primary neuronal cell cultures (**d**)

For our experiments, the HMC3 cell line was obtained from American Type Culture Collection (ATCC, VA, USA; CRL-3304™, Lot number: 70046457), which is responsible for its distribution and confirmation of authenticity through a comprehensive identification procedure. Although rodent microglia has been extensively studied, the use of human microglia has been limited until human immortalized microglial cell lines have been developed and incorporated in ATCC. HMC3 cells differ from the primary microglia, mainly due to SV40-dependent immortalization of human embryonic microglial cells. According to Rai et al. (2020) HMC3 express microglia-specific and also typical myeloid markers with the exception of low or absent CD45 and CD11b expression. However, the cells have been characterized as responding to a pattern of chemokines and inflammatory stimuli and also regulating the expression of activated microglia markers (Dello Russo et al. 2018; Baek et al. 2021). In accordance with the manufacturer’s instructions, cells were seeded in T-75 flasks and cultivated in 15 ml of complete growth medium consisting of EMEM supplemented with FBS (10%). Trypsinization with 0.25% (w/v) trypsin and 0.53 mM EDTA solution was performed after the cells reached 90% confluence. For the purpose of obtaining a homogenous population for the experiments, cells were subcultured twice, analyzed to exclude the presence of mycoplasma and then frozen in aliquots at passage 3 (p3). After thawing, the cells were moved from cryovial to T-75 flasks and suspended in EMEM supplemented with 10% FBS (Fig. 1a). Due to experimental plans requiring two types of the same media, differing only in the presence or absence of glucose, the next day it was necessary to replace the culture EMEM medium with DMEM, which is commercially available with and without glucose and has a composition similar to EMEM. The day before experiment, the cells were seeded on multiwell dishes (2 × 10^4^ viable cells per cm^2^) with reduced FBS concentration (5%) and allowed to recover overnight. The concentration of FBS was reduced in order to achieve more stable experimental conditions by controlling the proliferation rate, which is crucial for obtaining repeatable results.

### Primary neocortical neurons

Primary cultures of cortical neurons were obtained from mouse embryos at 15 days of gestation as previously described (Kajta et al. 2004). All animals were maintained according to the 3Rs principles and in compliance with European Union Legislation approved by the Bioethics Commission. Cortices from Swiss CD1 mouse embryos were dissected and digested with trypsin. After centrifugation, the isolated cells were suspended in Neurobasal medium supplemented with 10% FBS and placed in poly-*L*-ornithine-coated multiwell dishes at a density of ~ 2.0 × 10^5^ viable cells/cm^2^. After 3 days, the culture medium was exchanged for supplemented medium without FBS, and the cells were cultivated in a humidified atmosphere (37°C with 5% (vol/vol) CO_2_) for the next 4 days prior to the experiment. The percentage of astrocytes (identified with GFAP) did not exceed 10%, as previously described (Kajta et al. 2004).

### Experimental Models of Hypoxia/Ischemia

#### Hypoxia of HMC3 Cells and Primary Neurons

To simulate hypoxic conditions and obtain O_2_ levels close to zero, HMC3 cells and primary neurons were placed in a prewarmed and humidified hypoxia modular incubator chamber with 95% N_2_/5% CO_2_, as previously described (Wnuk et al. 2021a, Wnuk, Przepiórska et al. 2021). The O_2_ level in the chamber reached less than 0.5%, as measured with an oxygen analyzer. As for HMC3 cells, the experimental model consisted of the following steps: i. 6 h of hypoxia with a reduced concentration of FBS (1%) in DMEM (4.5 g/l glucose), ii. 18 h of reoxygenation with the restored concentration of FBS (5%) in the same DMEM (6 + 18 paradigm; Fig. 1b). To capture hard to observe effects related to caspase-1 activity, we decided to shorten the chosen paradigm to 2 h of hypoxia and 5 h of reoxygenation, but only in this one case (2 + 5 paradigm; Fig. 1c). For primary neuronal cell cultures, the experimental model consisted of the following steps: i. 6 h of hypoxia in neurobasal medium (4.5 g/l glucose), ii. 18 h of reoxygenation with replaced neurobasal medium (Fig. 1d).

#### Ischemia of HMC3 Cells and Primary Neurons

To mimic ischemic conditions and deprive cells of O_2_ and glucose, HMC3 cells and primary neurons were placed in a prewarmed and humidified hypoxia modular incubator chamber with 95% N_2_/5% CO_2_ in glucose-free medium. As for HMC3 the following experimental procedure was used: i. 6 h of ischemia with a reduced concentration of FBS (1%) in DMEM without glucose (0 g/l), ii. 18 h of reoxygenation with the restored concentration of FBS (5%) in DMEM supplemented with 4.5 g/l glucose (6 + 18 paradigm; Fig. 1b). Only in the case of caspase-1 activity assessment, we decided to shorten the chosen paradigm to 2 h of ischemia and 5 h of reoxygenation to capture hard to observe effects (2 + 5 paradigm; Fig. 1c). For primary neuronal cultures, the experimental model consisted of the following steps: i. 6 h of ischemia with neurobasal medium without glucose (0 g/l), ii. 18 h of reoxygenation with the medium replaced with standard neurobasal containing 4.5 g/l glucose (Fig. 1d). The control group included cultures that were not subjected to hypoxia or ischemia but were exposed to changes in FBS concentration analogous to those in the other groups (normoxic group).

### Post-treatment with Amorfrutin B

#### Treatment of HMC3 Cells Subjected to Hypoxia/Ischemia

In our study, we applied the post-treatment paradigm based on the application of amorfrutin B at the beginning of the reoxygenation period. This paradigm better reflects clinical standards due to the narrow therapeutic window of currently available therapies (up to 4.5 h). Taking into account our previous and present studies, we decided to apply 1 and 5 µM amorfrutin B, which induces neuroprotective effects in neurons subjected to hypoxia/ischemia. We wanted to observe specific effects on microglia to compare and follow-up effects obtained in our previous and present studies. Amorfrutin B was dissolved in DMSO and then in DMEM, resulting in DMSO concentrations less than 0.1%. After the experiments, the biological material was collected and used for further biochemical and molecular analyses.

#### Treatment of Primary Neurons Exposed to Hypoxia/Ischemia

To complement and compare the effects obtained in HMC3 cells, we decided to conduct additional experiments supporting our previous studies. Both concentration-dependent experiments (0.1, 1, 5, and 10 µM) and experiments with the most effective concentrations of amorfrutin B (1 and 5 µM) were performed. In pursuit of better clinical translation, the post-treatment paradigm was applied, i.e., amorfrutin B was added to the cell cultures at the beginning of the reoxygenation period for the next 18 h until the end of the experiments. Treated neurons were maintained in a humidified incubator (37 °C with 5% (vol/vol) CO_2_), and after 24 h of experiment, the biological material was collected for further biochemical or molecular analyses. After amorfrutin B was dissolved in DMSO, it was added to the neurobasal medium. The concentration of DMSO was less than 0.1%.

### Treatment with a PPARγ Antagonist (GW9662)

The involvement of the PPARγ receptor in amorfrutin B-induced neuroprotection was verified with the receptor antagonist GW9662 (1 µM). GW9662 was added to the culture medium after 6 h of hypoxia/ischemia in a post-treatment paradigm. After 40 min, amorfrutin B (1 and 5 µM) was added to the cell cultures for the next 18 h of reoxygenation. Amorfrutin B and GW9662 were used at concentrations that did not affect the level of cell neurodegeneration, as determined by Fluoro-Jade C; moreover, we applied both effective concentrations of amorfrutin B (1 and 5 µM) to avoid nonspecific effects. Both compounds were dissolved in DMSO and subsequently in culture medium, leading to a DMSO concentration less than 0.1%.

### Measurement of Cell Viability with AlamarBlue™ Reagent

After 6 h of hypoxia/ischemia and 18 h of amorfrutin B treatment, we diluted 10 × alamarBlue™ in cell culture medium and added it to the neuronal cultures. The cells were incubated with the solution for 3 h at 37 °C, and the absorbance was assessed at a wavelength of 570 nm (using 600 nm as a reference) with an Infinite M200 PRO microplate reader (Tecan, Mannedorf, Switzerland) and i-control software. The presence of viable cells induces a reduction in the color of blue resazurin to pink resorufin, and the absorbance level is proportional to the number of living cells (Lescat et al. 2019). All the results were compared with the absorbance levels of vehicle-treated cells and are presented as a percentage of the control ± SEM.

### Assessment of Neurodegeneration with Fluoro-Jade C

To assess the level of degenerating neurons in response to hypoxia/ischemia and amorfrutin B post-treatment, we decided to use Fluoro-Jade C (Wnuk et al. 2021a, Wnuk, Przepiórska et al. 2021; Pietrzak-Wawrzyńska et al. 2023). This fluorochrome dye was diluted in distilled water, resulting in a 0.005% working solution. After the experiment, 100 µl of culture medium was replaced with Fluoro-Jade C reagent per well, and the cells were incubated for 1 h. Subsequently, the fluorescence was measured at an excitation wavelength = 490 nm and an emission wavelength = 525 nm using an Infinite M200 PRO microplate reader, and i-control software. The fluorescence data were compared to those of the vehicle-treated cells and are presented as a percentage of the control ± SEM. Fluoro-Jade C is a green fluorescent dye, and the fluorescence intensity is proportional to the number of degenerating neurons.

### Immunofluorescence Staining with IBA1

Immunofluorescence staining with IBA1 and confocal microscopy were used to detect and visualize microglia, which were cultured on glass coverslips. After 6 h of hypoxia/ischemia and 18 h of amorfrutin B treatment, the cultures were fixed with 4% paraformaldehyde. After 3 washes, the cells were incubated for 1 h with blocking buffer containing 5% normal donkey serum and 0.3% Triton X-100 in 0.01 M PBS. Then, the microglial cells were treated with an anti-IBA1 primary antibody (diluted 1:100) and incubated at 4 °C for 24 h. The last step of the assay involved incubating the cells with a secondary antibody, after which the slides were washed, mounted, and coverslipped. The negative control with blocking buffer depleted of primary antibody excluded the nonspecific binding of the secondary antibody to the sample components and confirmed the specific binding of the IBA1 to its target antigen. The microscopic preparations were analyzed with a Leica TCS SP8 WLL confocal laser scanning microscope (DMi8-CS, Leica Microsystems, Wetzlar, Germany), and the pixel intensity was assessed using ImageJ software (FIJI version; 1.54f). The frequency of the brightest pixels in the region of interest was quantified by determining the mean fluorescence intensity excluding the background. The immunofluorescence signal corresponded to the IBA1 expression level.

#### Estimation of Caspase-1 Activity

Caspase-1 activation was assessed in microglial cells exposed to hypoxia/ischemia and amorfrutin B post-treatment. Cultures were lysed with buffer containing DTT, and the cell lysates were incubated with caspase-1 substrate (Ac-Trp-Val-Ala-Asp-*p*NA; Sigma‒Aldrich, USA) at 37 °C. According to the manufacturer, this colorimetric substrate cleaves specifically at aspartic acid in the presence of caspase-1, and the level of released *p*-nitroaniline was determined by measuring the absorbance at 405 nm. After 1 h of incubation, the level of *p*-nitroaniline was measured with an Infinite M200 PRO microplate reader and i-control software. The results were compared to the absorbance of the vehicle-treated cells (normoxic conditions) and are presented as the percentage of the control ± SEM.

#### Assessment of the Mitochondrial Membrane Potential with JC-1

To determine the mitochondrial membrane potential in microglial cells subjected to hypoxia/ischemia and amorfrutin B post-treatment, the JC-1 was added to the plate and incubated for 1 h at 37 °C, as previously described (Rzemieniec et al. 2015; Wnuk et al. 2020). This lipophilic dye enters mitochondria, and in healthy cells, forms aggregates, which emit red fluorescence (an indicator of increased mitochondrial membrane potential). The JC-1 solution was replaced with PBS, and both red (540 nm/590 nm) and green (490 nm/525 nm) fluorescence intensities were measured with an Infinite M200 PRO microplate reader. The data are expressed as the red to green fluorescence ratio and are presented as a percentage of the control ± SEM.

#### Fluorescence Staining with Calcein AM and Hoechst 33342

Microglial cell cultures were subjected to hypoxia/ischemia and amorfrutin B post-treatment. After the experiment, the cells were incubated with Hoechst 33342 (2 µg/ml solution) at room temperature for 5 min. Then, the cultures were incubated with 2 µM calcein AM reagent at room temperature for 10 min, as previously described (Kajta et al. 2019). The cells were visualized with a Leica DM IL LED Inverted Microscope (Leica Microsystems, Wetzlar, Germany) using constant illumination settings. The living cells presented green cytoplasm (calcein AM), while blue fluorescence staining was specific for the nuclei of microglial cells (Hoechst 33342).

#### Microglial Morphometric Analysis

The total number of cells was estimated basing on the number of cell nuclei (Hoechst 33342), while morphometric analysis was performed basing on staining of living cells (calcein AM). The semi-automated analysis with ImageJ (Java-based FIJI version; 1.54f) was performed to assess: the ramification index, cell body area, minimum Feret diameter, and microglia cell number. The basic step of analysis involved converting the grayscale picture into a binary image with setting the threshold value. Segmenting the image and separating the foreground from the background constituted a crucial action in determining cell number and morphological status. Particle analysis provided valuable insights into the total number of particles in image (microglial cell counting), area occupied by each particle (cell body surface), and closest possible distance between the two parallel tangents of an object (minimum Feret diameter). The ramification index was determined by fractal analysis as presented in previous studies (Becker et al. 2018; Kogel et al. 2021; Wittekindt et al. 2022). It was calculated in three steps: i. estimation of the cell area (A_c_), ii. using convex hull algorithm to measure projection area (A_p_), iii. determining the ratio of A_c_ to A_p_ and interpretation of obtained results. Quiescent microglia exhibit small bodies and ramified processes (small A_c_ and large A_p_), while activated microglia is characterized by hypertrophy of soma and retracted processes (similar A_c_ and A_p_). Consequently, the ramification index of activated microglia approaches the value close to 1. An increased ramification index indicates morphological changes and heightened reactivity of microglia. The results of all analyses are presented as mean or as the percentage of the control ± SEM.

##### Measurement of MTT Reduction

A colorimetric MTT assay was used to evaluate the mitochondrial function and metabolic activity of microglial cells subjected to hypoxia/ischemia and amorfrutin B treatment. The cell cultures were treated with MTT solution, and after 1 h of incubation at 37 °C, the reagent was replaced with 100% DMSO, which was used to dissolve the formazan crystals. The MTT assay is based on reducing MTT by oxidoreductase enzymes to purple formazan, and the intensity of the purple color is proportional to the metabolic activity of the cells. The absorbance at 570 nm was measured with an Infinite M200 PRO microplate reader, and the data were analyzed with i-control software and are presented as a percentage of the control ± SEM.

##### Determination of LDH Release

A Cytotoxicity Detection Kit was used to assess the lactate dehydrogenase level in cell-free supernatants collected immediately after the experiment. The supernatants were incubated with the appropriate reaction mixture for 30–60 min, resulting in a colorimetric reaction, as previously described (Kajta et al. 2001). The intensity of the red color was measured using an Infinite M200 PRO microplate reader at a wavelength of 490 nm and was proportional to the extent of plasma membrane damage in response to hypoxia/ischemia. The results obtained from the experiments were analyzed with an i-control and are presented as a percentage of the control ± SEM.

##### Assessment of the Proliferation Potential with BrdU

To quantify the incorporation of BrdU into newly synthesized DNA from active proliferating microglial cells, a BrdU cell proliferation assay was applied after hypoxia/ischemia and amorfrutin B post-treatment, according to the manufacturer’s protocol. The cell medium was complemented with 20 µl of working stock of BrdU per well. After 2 h of incubation at 37°C, the reagent was removed, and the cells were reconstituted with fixative/denaturing solution. The next step involved incubating the cells with an anti-BrdU antibody (1:100 dilution), followed by exposure to a peroxidase-conjugated goat anti-mouse IgG HRP. The cells were washed 3 times with buffer and subjected to substrate solution, followed by stop solution. The absorbance of each well was measured at 450 nm with an Infinite M200 PRO microplate reader, and the data were analyzed with i-control software. The results are presented as a percentage of the control ± SEM.

##### qPCR Analysis of mRNAs Specific to the Genes Encoding *IL1B*, *IL10*, *TNFA*, *PPARG*, *PGC1A *and *BCL2*

Microglial cells were subjected to hypoxic/ischemic conditions and amorfrutin B post-treatment, after which total RNA was collected and isolated using an RNeasy Mini Kit (Qiagen, Hilden, Germany) as previously described (Wnuk et al. 2016, 2018). After spectrophotometric determination of the RNA content in the sample, reverse transcription, and quantitative polymerase chain reaction (qPCR) were performed using a CFX96 Real-Time PCR Detection System (Bio-Rad, Hercules, CA, USA). The RNA (1000 ng) was reverse-transcribed to the cDNA template in a final volume of 50 µl using a high-capacity cDNA reverse transcription kit. The obtained products were amplified using TaqMan Gene Expression Assays specific for genes encoding *IL1B*, *IL10*, *TNFA*, *PPARG*, *PGC1A*, and *BCL2*. The entire volume of the reaction mixture contained 10 μl of Fast Probe qPCR Master Mix, 8 μl of RNase-free water, 1 µl of cDNA, and 1 μl of TaqMan Gene Expression Assay. The qPCR process consisted of series of temperature changes: 2 min at 50 °C and 10 min at 95 °C, followed by 55 cycles of 15 s at 95 °C and 1 min at 60 °C. The mRNA expression level was detected in the 40^th^ (last) cycle; however, due to the small amount of material, we decided to increase the number of cycles from 40 to 55. The data were analyzed using the Ct for each sample and the delta Ct method. The reference gene was chosen from among *GAPDH*, *HPRT* and *ACTB*, while geNorm, NormFinder, and BestKeeper pointed to *ACTB* as the most stable reference gene. The obtained results are presented as fold changes ± SEM.

##### ELISAs for IL-1β, IL-10, PPARγ, PGC1α, and BCL2

After 24 h of experiment, the microglial cells were gently washed with ice-cold PBS and lysed with ice-cold RIPA lysis buffer supplemented with a protease inhibitor cocktail. The collected samples were sonicated and centrifuged (15,000 × g for 20 min at 4 °C), and the obtained supernatants were collected as previously described (Kajta et al. 2017; Rzemieniec et al. 2019). The protein concentration was estimated using the Bradford method, and bovine serum albumin was used as a standard. Both standards and samples of known concentrations (~ 2 µg/µl) were added to plates precoated with IL-1β, IL-10, PPARγ, PGC1α, and BCL2. Next, biotin-labeled detection antibodies and streptavidin-HRP were added to each well. All the wells were washed with buffer, and the reaction was completed by adding substrate solutions. The color of the reaction developed according to the concentration of the protein of interest, and the reaction was terminated by the addition of acidic stop solution. The absorbance was measured at 450 nm with an Infinite M200 PRO microplate reader, and the data are presented as a percentage of the control value ± SEM or pg/mg of total protein.

##### Data Analysis

All the results were obtained as the absorbance or fluorescence units per well for alamarBlue™, caspase-1, MTT, LDH, BrdU or Fluoro-Jade C, JC-1, fluorescence units for qPCR; and pg per mg of total protein for the ELISAs. The statistical analysis of the data and the estimation of significance were conducted with analysis of variance (ANOVA), and the post hoc Newman‒Keuls test. A *p* value less than 0.05 was considered to indicate statistical significance and is presented as follows: ^*^p < 0.05, ^**^p < 0.01, and ^***^p < 0.001 (compared to the control groups); ^#^*p* < 0.05, ^##^*p* < 0.01, and ^###^*p* < 0.001 (compared to the hypoxic group); ^^^*p* < 0.05, ^^^^*p* < 0.01, and ^^^^^*p* < 0.001 (compared to the ischemic group); and ^$$$^*p* < 0.001 (compared to the cells subjected to both hypoxia/ischemia and amorfrutin B post-treatment. The effects of amorfrutin B or GW9662 on selected basic parameters related to cell survival under normoxic conditions are presented in the supplementary information (Figs. S1 and S2, Tables S1 and S2). Differences in the effects of the hypoxic and ischemic models and effects of amorfrutin B action between the hypoxic and ischemic conditions are also presented as the supplementary material (Figs. S3–S10).

## Results

### Amorfrutin B Inhibited the Activation of Microglia Through Decrease in IBA1 Expression

**Fig. 2:**
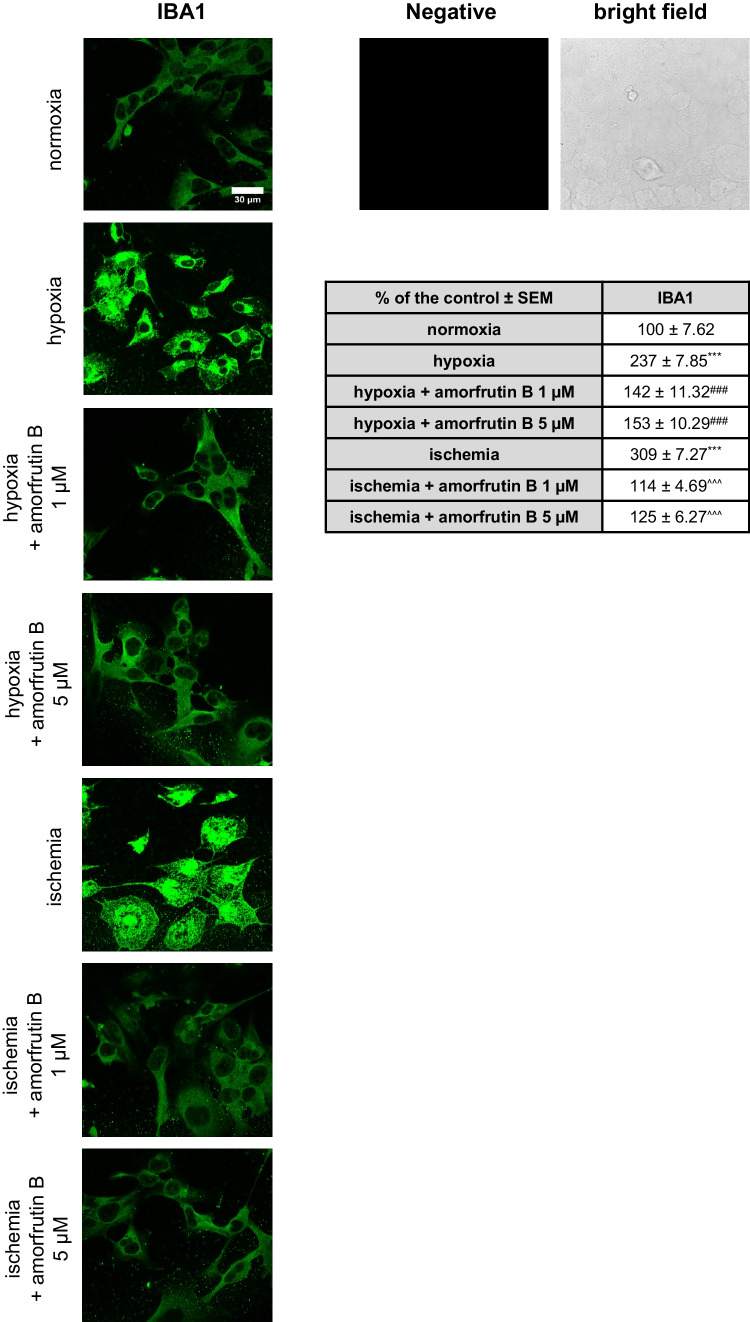
Post-treatment with amorfrutin B inhibited IBA1 expression (green staining). The negative control and the representative bright field image are also presented. The table shows the mean fluorescence intensity. The results are presented as a percentage of the control ± SEM. ^***^p < 0.001 compared to the control group; ^###^*p* < 0.001 compared to the cultures exposed to hypoxia; ^^^^^*p* < 0.001 compared to the cultures exposed to ischemia. There were 5 replicates in each group

In this study, a 6-h hypoxia/ischemia model followed by 18 h of reoxygenation/amorfrutin B treatment was used. Immunofluorescence staining and confocal microscopy confirmed the expression of IBA1 by microglia. IBA1-specific staining revealed that both during hypoxia and ischemia, there was an increase in signal intensity, which was reduced after amorfrutin B (1 and 5 µM) treatment (Fig. 2). The basic intensity quantification method indicated that during hypoxia, there was an increase in fluorescence intensity from 100% (normoxia) to 237%, while amorfrutin B decreased it to 142% (1 µM) and 153% (5 µM). In the case of ischemia, the fluorescence intensity reached 309%, and amorfrutin B post-treatment decreased the fluorescence intensity to 114% (1 µM) and 125% (5 µM). The negative control without primary antibody excluded the nonspecific binding of the secondary antibody to the sample. The IBA1 staining of microglial cells under normoxic conditions and amorfrutin B treatment was provided as supplementary material (Fig. S1).

### Post-treatment with Amorfrutin B Decreased Proinflammatory Caspase-1 Activity During Hypoxia and Ischemia

#### 6 h of Hypoxia/Ischemia Followed by 18 h of Reoxygenation with or without Amorfrutin B Post-treatment

**Fig. 3:**
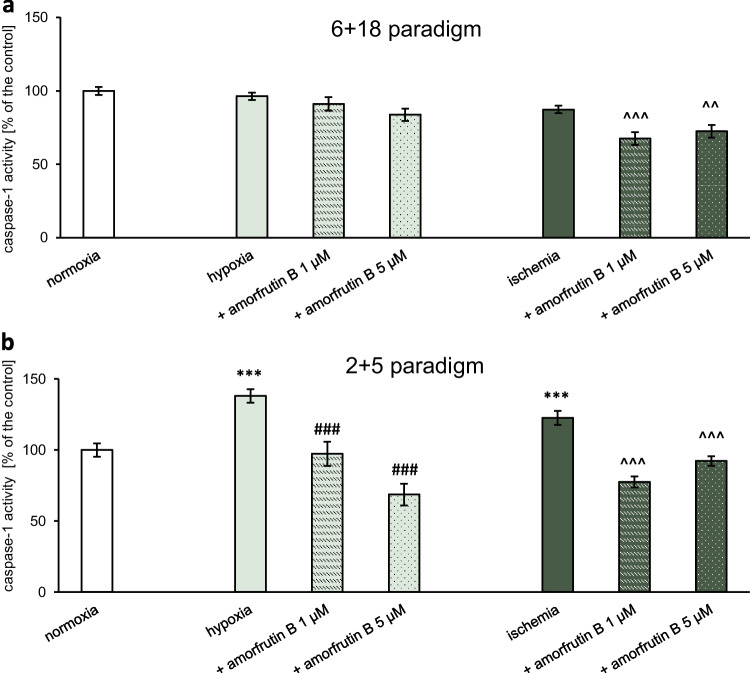
Amorfrutin B treatment after 6 h of ischemia inhibited caspase-1 activity in microglia (**a**), while administration of amorfrutin B after 2 h of hypoxia/ischemia reduced caspase-1 activity, which was increased in microglia in response to hypoxia and ischemia (**b**). The results are presented as a percentage of the control ± SEM of 3 independent experiments, consisting of 8–12 replicates per group. ^***^p < 0.001 compared to the control group; ^###^*p* < 0.001 compared to the cultures exposed to hypoxia; ^^^^*p* < 0.01, ^^^^^*p* < 0.001 compared to the cultures exposed to ischemia

During 6 h of hypoxia/ischemia and 18 h of reoxygenation, there were no changes in caspase-1 activity (96% and 87%, respectively), while amorfrutin B post-treatment decreased caspase-1 levels only under ischemic conditions (Fig. 3a). During ischemia, 1 and 5 µM amorfrutin B reduced caspase-1 activity to 68% and 72% of the control value, respectively.

#### 2 h of Hypoxia/Ischemia Followed by 5 h of Reoxygenation with or without Amorfrutin B Post-treatment

In our study, 2 h of hypoxia/ischemia and 5 h of reoxygenation increased caspase-1 activity up to 138% and 123% of the control value, respectively. Under hypoxic conditions, amorfrutin B at both concentrations (1 and 5 µM) reversed these effects to 97% and 67%, respectively. In the case of ischemia, amorfrutin B (1 and 5 µM) also decreased this parameter to 78% and 92% of the control value, respectively (Fig. 3b). The caspase-1 activities in microglial cells under normoxic conditions and amorfrutin B treatment (both paradigms) were provided as supplementary material (Table S1a).

### Administration of Amorfrutin B Inhibited Inflammation in Microglia Subjected to Hypoxia and Ischemia

#### Gene Expression of *IL1B*, *IL10*, and *TNFA* in Microglia

**Fig. 4:**
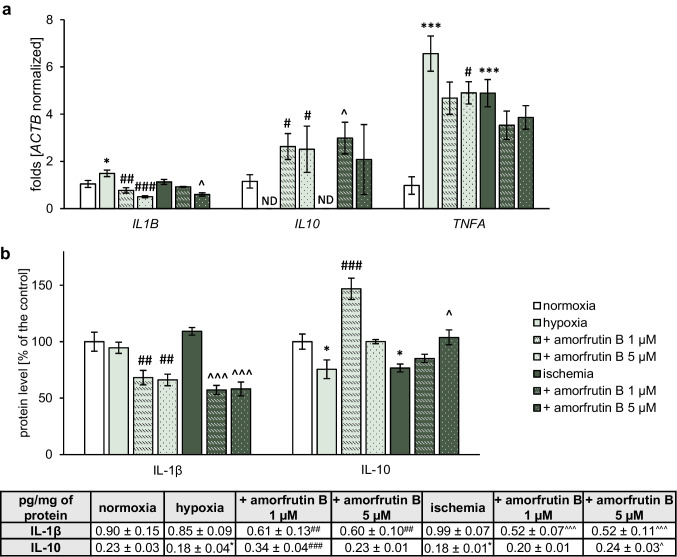
Post-treatment with amorfrutin B diminished the expression level of proinflammatory factors and induced/increased the expression level of anti-inflammatory factor in microglia subjected to hypoxia and ischemia. These changes are related to the expression levels of genes (**a**) and proteins (**b**). The obtained results are presented as the fold change in the case of qPCR and percentage of the control or pg/mg of protein ± SEM in the case of ELISA. There were 3 independent experiments, consisting of 5–6 replicates per group. ^*^p < 0.05 and ^***^p < 0.001 compared to the control group; ^#^*p* < 0.05, ^##^*p* < 0.01 and ^###^*p* < 0.001 compared to the cultures exposed to hypoxia; ^^^*p* < 0.05 and ^^^^^*p* < 0.001 compared to the cultures exposed to ischemia. ND indicates that the expression level was undetectable

The mRNA expression analysis with qPCR showed that cells exposed to 6 h of hypoxia/ischemia and 18 h of reoxygenation had increased expression levels of proinflammatory factors, such as *IL1B* (1.49-fold) during hypoxia and *TNFA* during hypoxia (6.57-fold), and ischemia (4.89-fold). In response to amorfrutin B post-treatment, *IL1B* expression was decreased to 0.77-fold (1 µM amorfrutin B), 0.50-fold (5 µM amorfrutin B) during hypoxia, and 0.60-fold (5 µM amorfrutin B) in the case of ischemia. Amorfrutin B at a concentration of 5 µM decreased *TNFA* expression to 4.90-fold only during hypoxia, while during ischemia, there were no statistically significant changes in *TNFA* expression (Fig. 4a). Interestingly, the *IL10* expression level was not detectable both during hypoxia or ischemia; however, amorfrutin B post-treatment restored the expression of this gene at the detectable level under hypoxic conditions (2.63-fold in the case of 1 µM amorfrutin B and 2.51-fold in the case of 5 µM amorfrutin B) and ischemic conditions (2.99-fold in the case of 1 µM amorfrutin B and 2.08-fold in the case of 5 µM amorfrutin B).

#### Protein Expression Levels of IL-1β and IL-10 in Microglia

ELISAs showed that the protein levels of IL-1β and IL-10 reached 0.90 and 0.23 pg/mg of total protein, respectively (Fig. 4b). Both under hypoxic and ischemic conditions, the protein level of IL-1β remained unchanged; however, post-treatment with 1 or 5 µM amorfrutin B reduced the level of this factor as follows: 0.61 pg/mg (equal to 68% of the control) and 0.60 pg/mg (equal to 66% of the control) in the hypoxic model, respectively, and 0.52 pg/mg in the case of both concentrations (equal to 57% of the control) in the ischemic model. Both hypoxia and ischemia reduced the protein level of IL-10 to 0.18 pg/mg (equal to 76% of the control). During hypoxia, post-treatment only with 1 µM amorfrutin B increased the IL-10 concentration to 0.34 pg/mg (equal to 147% of the control), while during ischemia, post-treatment only with 5 µM amorfrutin B increased the IL-10 concentration to 0.24 pg/mg (equal to 104% of the control).

### Amorfrutin B Increases the Expression of *PPARG*/PPARγ and PGC1α in Microglia Subjected to Hypoxia and Ischemia

#### Gene Expression of *PPARG* and *PGC1A* in Microglia

**Fig. 5:**
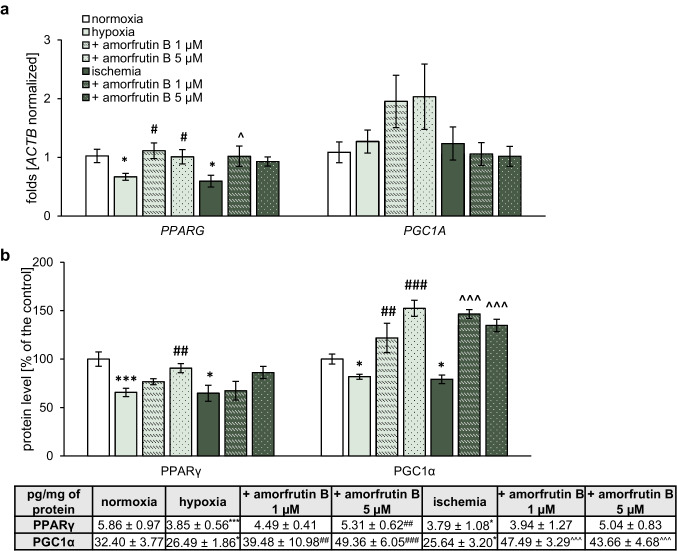
Amorfrutin B post-treatment increased *PPARG*/PPARγ and PGC1α protein in microglia under hypoxic/ischemic conditions. The results are presented as the fold change in the case of qPCR (**a**) and as the percentage of the control or pg/mg of protein ± SEM in the case of ELISA (**b**). There were 3 independent experiments, consisting of 5–6 replicates per group. ^*^p < 0.05 and ^***^p < 0.001 compared to the control group; ^#^*p* < 0.05, ^##^*p* < 0.01 and ^###^*p* < 0.001 compared to the cultures exposed to hypoxia; ^^^*p* < 0.05 and ^^^^^*p* < 0.001 compared to the cultures exposed to ischemia

In microglial cultures exposed to hypoxia and ischemia, the expression level of *PPARG* reached 0.67-fold and 0.60-fold, respectively. In the case of hypoxia, the administration of amorfrutin B (1 and 5 µM) at the beginning of the reoxygenation period resulted in an increase in *PPARG* expression to 1.11-fold and 1.01-fold relative to the control, respectively. In the case of ischemia, post-treatment with 1 µM amorfrutin B resulted in an increase in *PPARG* expression to 1.02-fold of the control. Neither hypoxia/ischemia nor amorfrutin B (1 or 5 µM) treatment affected *PGC1A* expression (Fig. 5a).

#### Protein Expression Levels of PPARγ and PGC1α in Microglia

In control cells, the protein levels of PPARγ and PGC1α reached 5.86 pg/mg and 32.40 pg/mg, respectively, as determined by ELISA (Fig. 5b). During hypoxia and ischemia, the protein level of PPARγ decreased to 3.85 pg/mg (equal to 66% of the control) and 3.79 pg/mg (equal to 65% of the control), respectively. Amorfrutin B partially reversed these effects only at a concentration of 5 µM, and during hypoxia, the PPARγ level reached 5.31 pg/mg (equal to 91% of the control). The protein level of PGC1α was diminished to 26.49 pg/mg (equal to 82% of the control) and 25.64 pg/mg (equal to 79% of the control) in response to hypoxia and ischemia, respectively. In the case of hypoxia, treatment with 1 or 5 µM amorfrutin B increased the PGC1α level up to 39.48 pg/mg (equal to 122% of the control) and 49.36 pg/mg (equal to 152% of the control), respectively. In the case of ischemia, treatment with 1 or 5 µM amorfrutin B increased the PGC1α level up to 47.49 pg/mg (equal to 147% of the control) and 43.66 pg/mg (equal to 135% of the control), respectively.

### Administration of Amorfrutin B Reduced the Mitochondrial Membrane Potential in Microglia Exposed to Hypoxia and Ischemia

**Fig. 6:**
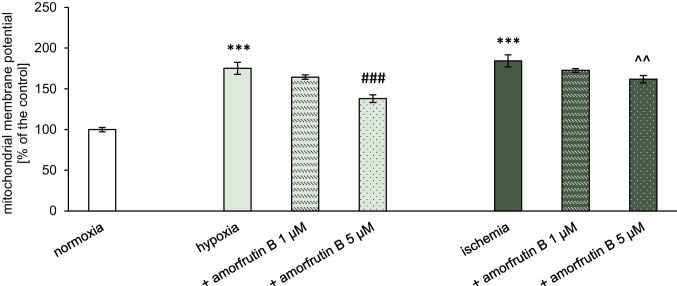
In microglia, post-treatment with amorfrutin B (5 µM) partially reversed hypoxia- and ischemia-induced increases in the mitochondrial membrane potential. The results are presented as a percentage of the control ± SEM of 3 independent experiments, consisting of 8–12 replicates per group. ^***^p < 0.001 compared to the control group; ^###^*p* < 0.001 compared to the cultures exposed to hypoxia; and ^^^^*p* < 0.01 compared to the cultures exposed to ischemia

Our study using JC-1 demonstrated that 6 h of hypoxia/ischemia and 18 h of reoxygenation led to increases in the mitochondrial membrane potential to 175% and 184% compared to normoxia, respectively (Fig. 6). Amorfrutin B at a concentration of 1 µM did not induce changes in mitochondrial membrane potential, but post-treatment with 5 µM amorfrutin B decreased it to 138% during hypoxia and 162% during ischemia. The mitochondrial membrane potential in microglial cells under normoxic conditions and amorfrutin B treatment was presented as supplementary material (Table S1b).

### Post-treatment with Amorfrutin B Reduced *BCL2*/BCL2 Expression in Microglia Subjected to Hypoxia/Ischemia

#### Gene Expression of *BCL2* in Microglia

**Fig. 7:**
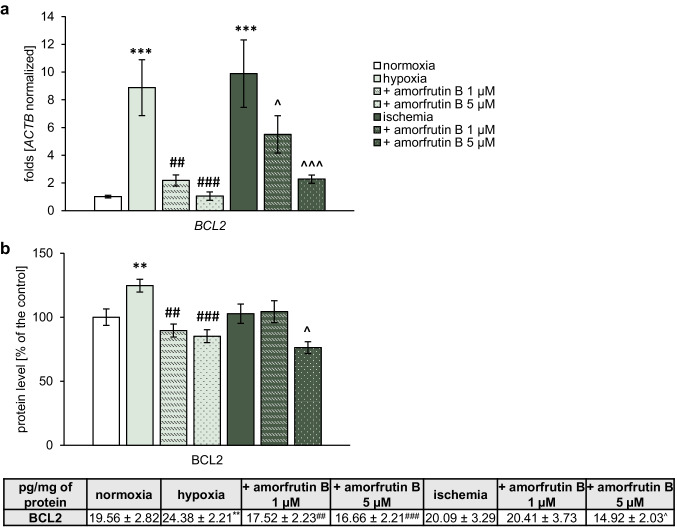
Administration of amorfrutin B after hypoxia/ischemia decreased the gene expression (**a**) and protein level (**b**) of *BCL2*/BCL2 in microglia. The results are presented as the fold change in the case of qPCR and as the percentage of the control or pg/mg of protein ± SEM in the case of ELISA. There were 3 independent experiments, consisting of 5–6 replicates per group. ^**^p < 0.01 and ^***^p < 0.001 compared to the control group; ^##^*p* < 0.01 and ^###^*p* < 0.001 compared to the cultures exposed to hypoxia; and ^^^*p* < 0.05 and ^^^^^*p* < 0.001 compared to the cultures exposed to ischemia

qPCR analysis revealed that both hypoxia and ischemia caused a massive increase in the expression level of *BCL2*, which reached 8.88-fold and 9.88-fold compared to normoxia, respectively (Fig. 7a). Amorfrutin B at both concentrations (1 and 5 µM) decreased the expression of *BCL2* during hypoxia, which was manifested as a decrease to 2.18-fold and 1.06-fold, respectively. As for ischemia, post-treatment with 1 or 5 µM amorfrutin B decreased the *BCL2* expression level to 5.51-fold or 2.28-fold, respectively.

#### Protein Expression Level of BCL2 in Microglia

ELISA indicated that hypoxia increased the protein level of BCL2 from 19.56 pg/mg (normoxia) to 24.38 pg/mg (equal to 125%), while post-treatment with 1 and 5 µM amorfrutin B reduced this level to 17.52 pg/mg (equal to 90% of the normoxic value) and 16.66 pg/mg (equal to 85% of the normoxic value), respectively. Ischemia did not affect the protein level of BCL2, however, post-treatment with 5 µM amorfrutin B decreased the protein level of BCL2 to 14.92 pg/mg, which was equal to 76% of the normoxic value, while a 1 µM concentration had no effect (Fig. 7b).

### Amorfrutin B Normalized the Morphology of Microglia During Hypoxia/Ischemia

**Fig. 8:**
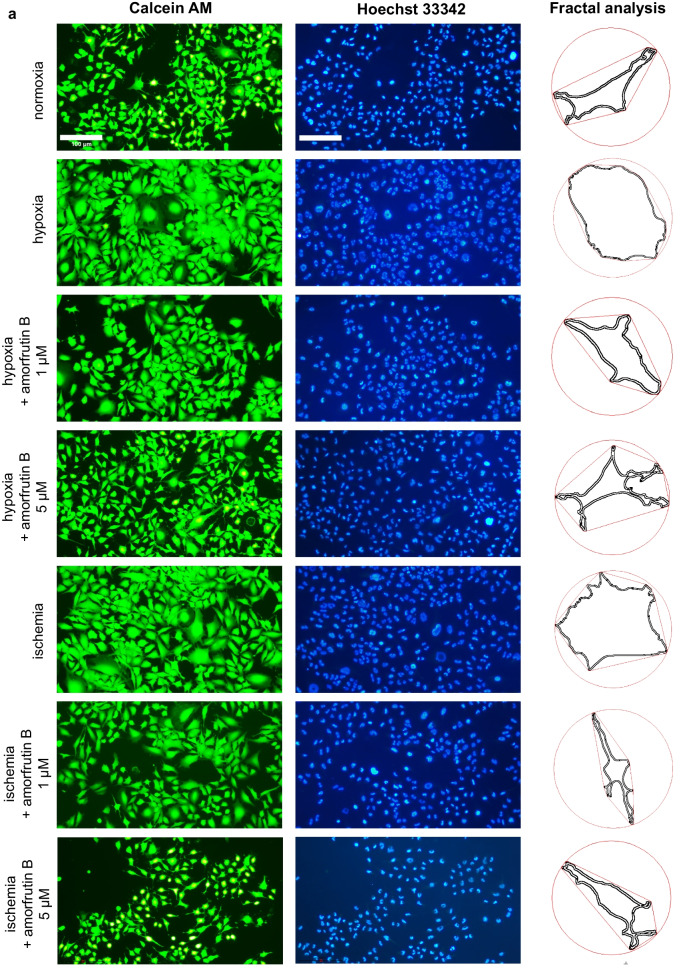

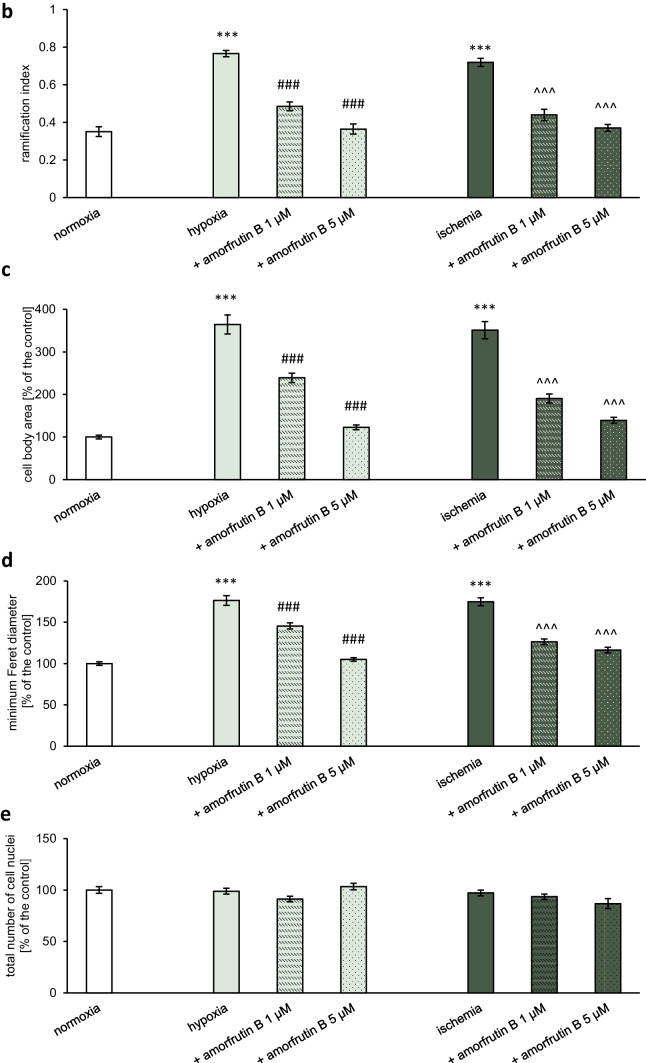
Fluorescence staining was used to visualize viable microglia using calcein AM (green labeling), and cell nuclei were visualized using Hoechst 33342 (blue labeling). Particle and fractal analysis enabled the morphometric analysis (**a**). Amorfrutin B post-treatment induced a shift in microglial morphology during hypoxia/ischemia from amoeboid-like to ramified cells (**b**) with smaller cell bodies (**c** and **d**). The number of microglial cell nuclei remained unchanged regardless of the conditions (**e**). ^***^p < 0.001 compared to the control group; ^###^*p* < 0.001 compared to the cultures exposed to hypoxia; ^^^^^*p* < 0.001 compared to the cultures exposed to ischemia. There were 10 replicates in each group

To examine the gross morphology of the HMC3 cells, we used calcein AM to label living cells and Hoechst 33342 to visualize the cell nuclei. The morphometric analysis of cells was conducted using particle and fractal analysis (Fig. 8a). In case of hypoxia the ramification index increased to 0.77, while amorfrutin B (1 and 5 µM) post-treatment reduced this value to 0.48 and 0.36, respectively. Ischemia also caused an increase in the ramification index, reaching value 0.72, while amorfrutin B decreased this value to 0.44 (1 µM amorfrutin B) and 0.37 (5 µM amorfrutin B). Comparing to normoxia (ramification index = 0.35), we can infer that values close to 1 indicated microglial cell activation, while amorfrutin B normalized this parameter under hypoxic and ischemic conditions, thereby restoring the morphology of cells characteristic of quiescent microglia (Fig. 8b). During hypoxia and ischemia, there was an increase in cell body area (up to 364% and 351%, respectively) and minimum Feret diameter (up to 176 and 175%, respectively). Treating cells with amorfrutin B (1 and 5 µM) after hypoxic injury resulted in a decrease in cell body surface to 239% and 123%, as well as decrease in minimum Feret diameter to 146% and 105%, respectively. Post-treatment with amorfrutin B (1 and 5 µM) after ischemia contributed to reduction in cell body surface to 191% and 139% (Fig. 8c), as well as reduction in minimum Feret diameter to 126% and 116%, respectively (Fig. 8d). The analysis of the number of cell nuclei (stained with Hoechst 33342) showed that it remained unchanged regardless of the conditions (Fig. 8e). Taking all together, this morphometric analysis indicated that 6 h of hypoxia/ischemia and 18 h of reoxygenation induced a morphological shift; the cells retracted their processes, taking on a rounded or amoeboid shape and large cell bodies (functionally M1-like phenotype). Post-treatment with amorfrutin B in both models resulted in morphological changes resembling the state of cells under normoxic conditions; i.e., cells exhibited a ramified appearance and had smaller cell bodies (functionally M2-like phenotype). The calcein AM and Hoechst 33342 staining of microglial cells under normoxic conditions and amorfrutin B treatment was provided as supplementary material (Fig. S2).

### Amorfrutin B Impaired the Hypoxia/Ischemia-induced Increase in Metabolic Activity of Microglia

**Fig. 9:**
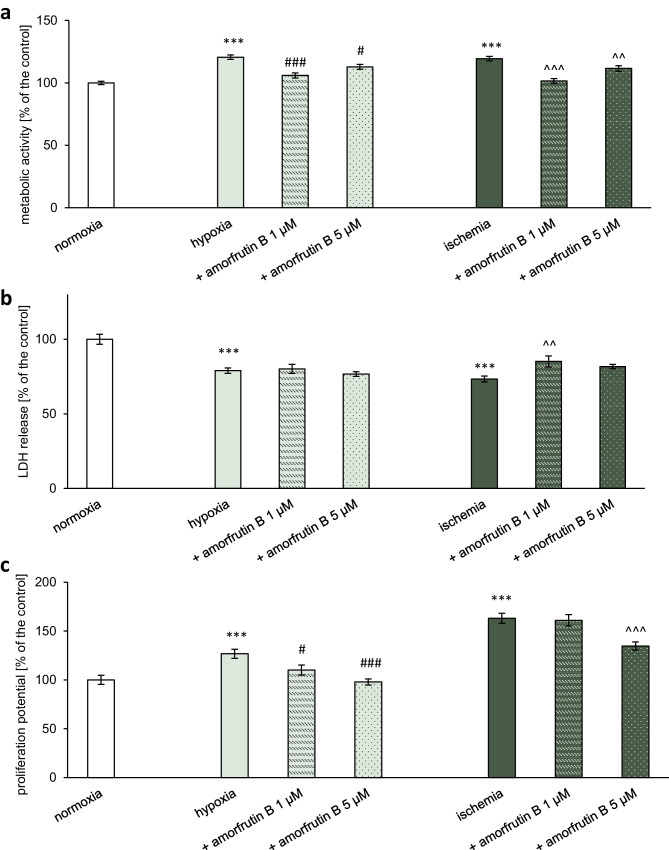
Post-treatment with amorfrutin B reversed the increase in metabolic activity (**a**) or decrease in LDH (**b**) and reduced the proliferation potential (**c**) of microglia exposed to hypoxia/ischemia. The results are presented as a percentage of the control ± SEM of 3 independent experiments, consisting of 8–12 replicates per group. ^***^p < 0.001 compared to the control group; ^#^*p* < 0.05 and ^###^*p* < 0.001 compared to the cultures exposed to hypoxia; and ^^^^*p* < 0.01 and ^^^^^*p* < 0.001 compared to the cultures exposed to ischemia

MTT assay determined that 6 h of hypoxic or ischemic conditions and 18 h of reoxygenation increased the metabolic activity of microglial cells from the control value (100%) to 121% during hypoxia and 119% during ischemia. In the hypoxic paradigm, post-treatment with amorfrutin B at both concentrations (1 and 5 µM) resulted in normalization of metabolic activity, which was manifested by decrease of this parameter to 106% and 112% of the control value, respectively. In the case of ischemia, amorfrutin B (1 and 5 µM) administration at the beginning of the reoxygenation period also resulted in metabolic activity normalization, manifested by decreases to 102% and 111% of the control value, respectively (Fig. 9a). The MTT reduction in microglial cells under normoxic conditions and amorfrutin B treatment was presented as supplementary material (Table S1c).

### Amorfrutin B Only Partially Reversed the Hypoxia/Ischemia-induced Decrease in LDH Release From Microglial Cells

Our study showed that 6 h of hypoxia or ischemia and 18 h of reoxygenation decreased LDH levels to 79% and 73% of the normoxic value, respectively. Exposure to 1 or 5 µM amorfrutin B for 18 h during the reoxygenation period after hypoxia did not affect LDH release. However, in response to 1 µM amorfrutin B during ischemic conditions, there was an increase in LDH to 85%, while 5 µM amorfrutin B did not affect this parameter (Fig. 9b). The LDH level in microglial cells under normoxic conditions and amorfrutin B treatment was presented as supplementary material (Table S1d).

### Amorfrutin B Reduced the Proliferation Potential of Microglia that was Induced in Response to Hypoxia/Ischemia

As demonstrated by the BrdU cell proliferation assay, hypoxia and ischemia induced the proliferative action of microglia, that was manifested as increase in proliferation potential level up to 127% and 163% of the normoxic value, respectively (Fig. 9c). In the case of hypoxia, administration of 1 or 5 µM amorfrutin B 6 h after injury resulted in a decrease in proliferation potential to 110% or 98% of the normoxic value, respectively. In the case of ischemia, only 5 µM amorfrutin B reduced the proliferation potential of microglia to 135% of the normoxic value, while 1 µM amorfrutin B was ineffective. The proliferation potential of microglial cells under normoxic conditions and amorfrutin B treatment was presented as supplementary material (Table S1e).

### Post-treatment with Amorfrutin B Improved the Viability of Neocortical Cell Cultures Subjected to Hypoxia and Ischemia

**Fig. 10:**
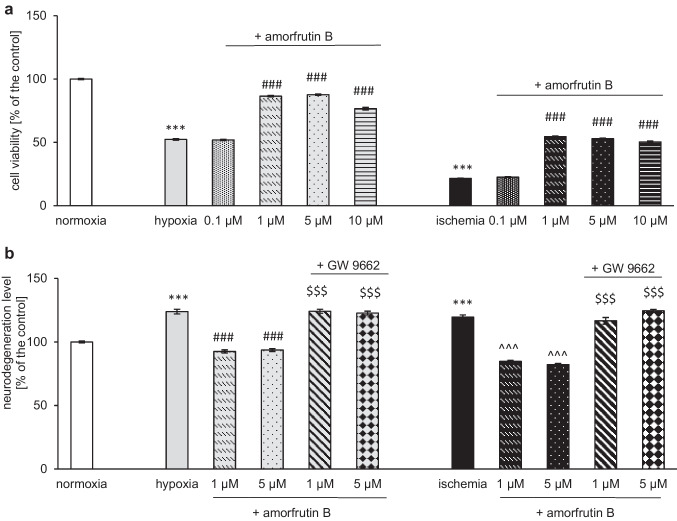
Amorfrutin B (1, 5 and 10 µM) post-treatment inhibited the hypoxia- and ischemia-dependent reductions in viability of neuronal cells, as indicated by alamarBlue™ (**a**). Administration of amorfrutin B (1 and 5 µM) reduced neurodegeneration, and the PPARγ antagonist (GW9662; 1 µM) reversed these effects, as indicated by Fluoro-Jade C (**b**). The results are presented as a percentage of the control ± SEM of 3 independent experiments, consisting of 8–12 replicates per group. ^***^p < 0.001 compared to the control group; ^###^*p* < 0.001 compared to the cultures exposed to hypoxia, ^^^^^*p* < 0.001 compared to the cultures exposed to ischemia; ^$$$^p < 0.001 compared to the cells subjected to both hypoxia/ischemia and amorfrutin B post-treatment

An alamarBlue™ assay was applied to assess the viability of primary neurons subjected to hypoxia/ischemia or amorfrutin B post-treatment. Our study showed that 6 h of hypoxia/ischemia and 18 h of reoxygenation resulted in a reduction in viability, which was manifested by a decrease in this parameter to 52% of the control and 22% of the control, respectively. The concentration-dependent responses to amorfrutin B post-treatment during hypoxia were as follows: 86% (1 µM amorfrutin B), 88% (5 µM amorfrutin B), and 77% (10 µM amorfrutin B) of the control value. In the case of ischemia, the concentration-dependent responses to amorfrutin B post-treatment were as follows: 55% (1 µM amorfrutin B), 53% (5 µM amorfrutin B), and 50% (10 µM amorfrutin B) of the control value. Administration of amorfrutin B at a concentration of 0.1 µM did not change the viability of neurons subjected to hypoxia or ischemia. In control cells (normoxic conditions), amorfrutin B treatment at concentrations ranging from 0.1 to 10 μM for 18 h did not affect the viability (supplementary materials, Table S2a). The most profound neuroprotective effects were obtained after the use of 1 or 5 μM amorfrutin B, and for that reason, these concentrations were selected for experiments conducted on human microglia (Fig. 10a).

### Amorfrutin B Post-treatment Reduced Hypoxia/Ischemia-induced Neurodegeneration, and the PPARγ Antagonist Reversed this Effect

Fluoro-Jade C labeling was used to determine the degree of degeneration of neuronal cells exposed to 6 h of hypoxia/ischemia and 18 h of reoxygenation/amorfrutin B post-treatment. The neurodegeneration level in response to hypoxia reached 124% of the control value, while post-treatment with 1 or 5 µM amorfrutin B reduced this parameter to 93% and 94% of the control, respectively. Simultaneous administration of GW9662 (1 µM, PPARγ antagonist) reversed the neuroprotective effects of amorfrutin B (1 and 5 µM), manifested as an increase in neurodegeneration up to 124% and 123% of the control value, respectively. Ischemia increased neurodegeneration up to 120%, while amorfrutin B post-treatment (1 and 5 µM) reduced this effect to 85% and 82% of the control value, respectively. Simultaneous treatment with 1 µM GW9662 reversed the neuroprotective effects of 1 and 5 µM amorfrutin B, that was manifested as enhancements in neurodegeneration level up to 117% and 124% of the control value, respectively (Fig. 10b). The neurodegeneration level under normoxic conditions and amorfrutin B treatment (given separately or in combination with GW9662) was presented as supplementary material (Table S2b).

## Discussion

Here, we provide evidence for specific amorfrutin B-induced effects on human microglia subjected to hypoxia/ischemia; this compound counteracts inflammation and influences mitochondrial status, metabolic activity and proliferation potential in a PPARγ-dependent manner. Amorfrutin B is an SPPARγM that we recently demonstrated for the first time to be a promising neuroprotective compound in cellular models of stroke and perinatal asphyxia. Indeed, we showed that post-treatment of mouse neurons with amorfrutin B caused neuroprotection that relied on its inhibitory action on hypoxia/ischemia-induced apoptosis, autophagy and oxidative stress as well as on normalization of the epigenetic status of neuronal cells (Wnuk et al. 2021a, Wnuk, Przepiórska et al. 2021; Przepiórska et al. 2023). Although neuronal mechanisms of amorfrutin B-evoked neuroprotection have been identified, none of them reflects the action of the compound on microglia known to play a pivotal role in the response of brain tissue to hypoxic and ischemic damage.

In the present study, we showed that in human microglial cell (HMC3) cultures subjected to hypoxia/ischemia, post-treatment with amorfrutin B decreased the IBA1 fluorescence intensity, which was accompanied by reduced caspase-1 activity and downregulated mRNA expression of *IL1B* and *TNFA* but not *IL10,* which was upregulated. We observed that the changes in the mRNA expression of interleukins were paralleled by changes in protein levels. IBA1 is an actin cross-linking protein that participates in the membrane ruffling of microglia. Its increased density is a measure of microglial activation, which is also manifested by overstimulated caspase-1 activity and elevated levels of interleukins in the brain during ischemic stroke (Li et al. 2019; Wittekindt et al. 2022). Importantly, post-mortem studies of human ischemic brains have shown that glial cells express higher levels of cytokines, including IL-1β and TNF-α (Clausen et al. 2020). Recently, Li et al. (2019) demonstrated that the inhibition of caspase-1 and the downregulation of proinflammatory factors in activated microglia ameliorated cerebral injury and reduced infarct volume in mice subjected to middle cerebral artery occlusion (MCAO). Therefore, one can assume that the ability to inhibit microglia-dependent inflammation is one of the key elements that constitute the therapeutic capacity of amorfrutin B against cerebral hypoxia/ischemia, as evidenced by our studies on human microglia.

In this study, we also demonstrated that post-treatment of human microglia subjected to hypoxia/ischemia with amorfrutin B stimulated PPARγ signaling, as evidenced by increased mRNA and/or protein levels of PPARγ and PGC1α. PPARγ is present in microglia, and its expression is downregulated during hypoxic/ischemic episodes (Xia et al. 2015). PPARγ has been defined as a predominant contributor to the anti-inflammatory profile of microglia, and its activation inhibits proinflammatory cytokine production in 3-nitropropionic acid-activated microglia and shifts macrophage polarization from the M1-like phenotype to the M2-like phenotype in obesity-induced inflammation in liver and adipose tissues (Mansour et al. 2022). Moreover, PPARγ was shown to prime human monocytes into macrophages with anti-inflammatory properties (Bouhlel et al. 2007). As for PGC1α, it has been categorized as a marker of ischemic stroke in humans, and microglia-specific overexpression of PGC1α significantly reduces proinflammatory cytokine production in the mouse brain after ischemic injury (Han et al. 2021). Since amorfrutin B is a selective PPARγ modulator, we postulate that its anti-inflammatory properties rely on PPARγ/PGC1α targeting and reprofiling of microglia to inhibit proinflammatory cytokines such as IL-1β and TNF-α and stimulate anti-inflammatory cytokines such as IL-10, as observed in the present study. Except for studies on the anti-inflammatory action of amorfrutin B on liver cells exposed to a high-fat diet (Weidner et al. 2013), there are no other data showing the anti-inflammatory potential of amorfrutin B in human microglia subjected to hypoxia/ischemia to compare our results with.

We also showed that amorfrutin B reversed the hypoxia/ischemia-evoked effects on mitochondria-related parameters, such as the mitochondrial membrane potential, *BCL2*/BCL2 expression and mitochondrial enzyme activity/metabolic activity, which were correlated with diminished proliferation potential of human microglia. In the LPS-induced inflammation model, it has been demonstrated that the mitochondrial membrane potential decreases in the initial stages but gradually returns to normal starting 3 h after LPS treatment, ultimately exceeding the baseline level by 6 h (Bauerfeld et al. 2012). The timeline of recovery and enhancement of mitochondrial potential after LPS treatment is similar to the timeline observed in our study paradigm after exposure of human microglia to hypoxia/ischemia. High mitochondrial membrane potential was also exhibited by microglia during chronic phase of experimental encephalomyelitis (C-EAE), which was associated with downregulation of mitophagy-related genes, increased activity of mitochondrial complexes I (CI) and II (CII) and reduced ATP levels (Peruzzotti-Jametti et al. 2024). This metabolic reprogramming was interpreted as promoting increased reactive oxygen species (ROS) generation, mainly through CI and reverse electron transport. In microglia, hypoxic/ischemic episodes are known to dysregulate the mitochondrial membrane potential and, in this way, increase metabolic activity; these changes are considered as indicators of activated microglia and were also observed in our studies. Since amorfrutin B reduces the mitochondrial membrane potential and metabolic activity in hypoxia/ischemia-challenged microglia, it can be assumed that this compound has property to control the activity of the CI and CII mitochondrial complexes and in this way may inhibit ROS generation in activated microglia. However, the only relevant study to compare our results with has been performed on a mouse microglia-like BV2 cell line exposed to LPS and telmisartan, which in contrast to amorfrutin B targets not only PPARγ, but also PPARα (Elkahloun et al. 2019). In the cited study, telmisartan was shown to normalize the expression of genes associated with mitochondrial activity (e.g., *Slc25a51*), which supports the effects of amorfrutin B observed in our study in terms of metabolic activity.

BCL2 is widely recognized as a mitochondrial protein, and its function is usually related to apoptosis and/or autophagy (Wnuk and Kajta 2017). Intriguingly, upregulation of anti-apoptotic BCL2 has been detected in Alzheimer’s and Huntington’s diseases patients, as well as in models of brain hypoxia and ischemia (Satou et al. 1995; Karlnoski et al. 2007; Sassone et al. 2013; Wnuk et al. 2020, 2021a, Wnuk, Przepiórska et al. 2021; Przepiórska et al. 2023; Pietrzak et al. 2023). Since adult human brain express more BCL2 in microglia than in neurons (Merry et al. 1994), and hypoxia/ischemia increase BCL2 expression in brain tissue (Chen et al. 1997), one can assume that this increase is dependent mainly on microglia-localized BCL2. We have previously shown that treatment with amorfrutin B increased BCL2 protein level in mouse neuronal cells undergoing hypoxia/ischemia, suggesting an anti-apoptotic effect of the compound on brain neurons (Przepiórska et al. 2023). In the present study, treatment of human microglial cells with amorfrutin B decreased *BCL2*/BCL2 mRNA and protein levels, suggesting that the compound affected BCL2-dependent processes and in this way controlled the activation of microglia. It became evident that, in addition to anti-apoptotic property, BCL2 plays a novel, noncanonical role in augmenting mitochondrial respiration and cytochrome c oxidase (COX) activity, which is closely linked to the slight pro-oxidant activity of the protein. High BCL2 expression level and increased COX activity have been reported in parallel with increased metabolic and mitochondrial activity, indicating the importance of BCL2-COX interaction and association with oxidative stress (Chen and Pervaiz 2010). BCL2 overexpression in lymphoid and myeloid lineages appeared to significantly change expression of genes associated with metabolic activity, cell cycle, immune response and apoptosis-related genes. Knowing that amorfrutin B reduces *BCL2*/BCL2 expression, accompanied by reduced metabolic activity and reduced proliferation potential of human microglia, it can be suggested that the mechanisms of action of amorfrutin B during hypoxia/ischemia include a decrease in mitochondrial respiration and microglial COX activity. Given that the microglial population is subject to continuous and rapid remodeling (Askew et al. 2017), amorfrutin B may shift the dynamic balance between microglial proliferation and microglial apoptosis.

In addition to providing evidence for the anti-inflammatory and PPARγ-dependent properties of amorfrutin B, as well as its ability to normalize mitochondrial status of microglial cells, we demonstrate for the first time that amorfrutin B has the ability to control the metabolic activity and proliferation potential of hypoxia/ischemia-activated microglia. Studies of Sapkota et al. (2017) characterized activated microglia as cells that exhibited increased intensity of IBA1/BrdU labeling, indicating enhanced microglial proliferation potential in the penumbra of MCAO mice. Similarly, Taylor and Sansing (2013) reported that activated microglia retract their ramifications and become amoeboid-like cells. In our study, particle and fractal analysis showed that hypoxia and ischemia increased ramification index, cell body area and minimum Feret diameter, and amorfrutin B reversed all these effects in a concentration-dependent manner. An increased ramification index indicates morphological changes and heightened reactivity of microglia subjected to hypoxia/ischemia. According to Hoechst 33342 staining, which was performed in parallel with calcein AM staining, the number of microglial cell nuclei did not change significantly in response to hypoxia/ischemia or amorfrutin B treatment. Considering the proliferation potential of microglial cells (BrdU incorporation) and metabolic activity (MTT assay), which were stimulated by hypoxia/ischemia and inhibited by amorfrutin B, it can be assumed that amorfrutin B impairs both mitochondrial function and the self-renewal potential of microglial cells. Therefore, stimulatory effect of amorfrutin B on microglia-dependent toxicity (LDH release) is the result of impaired function rather than impaired cell viability of microglia. Our data suggest that the properties of amorfrutin B to control metabolic activity and proliferation potential are restricted to hypoxia/ischemia-activated microglia, because amorfrutin B induced a shift in microglial morphology from amoeboid (M1-like) to ramified (M2-like), resembling the state of cells under normoxic conditions, i.e., cells with a branched appearance and smaller cell bodies.

To address the possible differences in the mechanisms of action of amorfrutin B between microglia and neurons subjected to hypoxia/ischemia, we performed additional experiments on mouse primary neurons. Current study complements our previous reports on neuroprotective capacity of amorfrutin B by unraveling strong stimulatory effect on hypoxia/ischemia-impaired neuronal cell viability and identifying a PPARγ-mediated decrease in neuronal degeneration. AlamarBlue™ cell viability indicator allowed neuronal cells to remain fully functional, viable and healthy, which is particularly important for cells exposed to hypoxia/ischemia and significantly outperforms analyzes based on the presence of the indicator in cells and causing their death (Longhin et al. 2022). According to the results, amorfrutin B promoted neuronal cell survival, as evidenced by increased cell viability (alamarBlue™) and reduced neurodegeneration (Fluoro-Jade C), which was achieved in a PPARγ-specific manner i.e., GW9662 reversed the amorfrutin B-evoked decrease in neuronal degeneration. Interestingly, the stimulatory effect of amorfrutin B on mouse neuronal survival, is opposite to the inhibitory effect of amorfrutin B on the proliferation potential and metabolic activity/mitochondrial function of human microglia. However, it should be emphasized that the compared cells differ in many features and the same analyzes may not sufficiently take into account the specificity of their response to hypoxia/ischemia.

In summary, this study showed for the first time that amorfrutin B compromises hypoxia/ischemia-induced activation of human microglia in a PPARγ-dependent manner, which involves inhibiting inflammation, normalizing mitochondrial status, and controlling metabolic activity and proliferation potential. These data extend the protective potential of amorfrutin B in the pharmacotherapy of hypoxic/ischemic brain injury, targeting not only neurons but also activated microglia.

## Supplementary Information

Below is the link to the electronic supplementary material.

Supplementary file1 (DOCX 3.04 MB)

## Data Availability

The datasets generated during and/or analyzed during the current study are available from the corresponding author on reasonable request.

## Abbreviations

*ACTB*: Actin Beta
ATP: Adenosine Triphosphate
*BCL2*/BCL2: B-cell lymphoma 2 (apoptosis regulator BCL2)
BrdU: Bromodeoxyuridine
C-EAE: Chronic experimental autoimmune encephalomyelitis
CI and CII: Mitochondrial complexes I and II
COX: Cytochrome c oxidase
Ct: Threshold cycle
DMEM: Dulbecco’s Modified Eagle’s Medium
DMSO: Dimethyl Sulfoxide
DTT: Ditiotreitol
EDTA: Ethylenediaminetetraacetic Acid
ELISA: Enzyme-linked Immunosorbent Assay
EMEM: Eagle's Minimum Essential Medium
FBS: Fetal Bovine Serum
GFAP: Glial Fibrillary Acidic Protein
HMC3: Human Microglial Clone 3 cell line
HSP60: Heat Shock Protein 60
IBA1: Ionized Calcium-binding Adaptor Molecule 1
*IL-10*/IL-10: Interleukin 10
*IL-1B*/IL-1β: Interleukin 1 beta
LDH: Lactate Dehydrogenase
LPS: Lipopolysaccharides
MCAO: Middle Cerebral Artery Occlusion
MTT: Thiazolyl Blue Tetrazolium Bromide
*PGC1A*/PGC1α: Peroxisome Proliferator Activated Receptor Gamma Coactivator-1 alpha
PPARγ: Peroxisome Proliferator Activated Receptor Gamma
qPCR: Quantitative Polymerase Chain Reaction
RIPA: Radioimmunoprecipitation Assay
ROS: Reactive oxygen species
rt-PA: Recombinant Tissue Plasminogen Activator
SEM: Standard Error of the Mean
SPPARγM: Selective PPARγ Modulator
SV40: Simian virus 40
*TNFA*/TNFα: Tumor Necrosis Factor Alpha
TZDs: Thiazolidinediones
