# Supplementary material for: Amorfrutin B Compromises Hypoxia/Ischemia-induced Activation of Human Microglia in a PPARγ-dependent Manner: Effects on Inflammation, Proliferation Potential, and Mitochondrial Status
Source: J Neuroimmune Pharmacol. 2024 Jul 1;19(1):34. doi: 10.1007/s11481-024-10135-9 (PMC11217078; doi:10.1007/s11481-024-10135-9)
Supplement: Supplementary file 1 — Supplementary file1 (DOCX 3.04 MB) [file 11481_2024_10135_MOESM1_ESM.docx]

**Supplementary information**

**Figure S1.** Administration of 1 and 5 µM amorfrutin B did not affect IBA1 expression and morphology of microglial cells under normoxic conditions, respectively

- normoxia





- 1 µM amorfrutin B





- 5 µM amorfrutin B





| % of the control ± SEM | **IBA1** |
| --- | --- |
| normoxia | 100 ± 9.18 |
| normoxia + amorfrutin B 1 µM | 108 ± 5.71 |
| normoxia + amorfrutin B 5 µM | 89 ± 13.48 |

**Figure S2.** Administration of 1 and 5 µM amorfrutin B did not affect the morphology of microglial cells under normoxic conditions, respectively, as indicated by calcein AM and Hoechst 33342

- normoxia


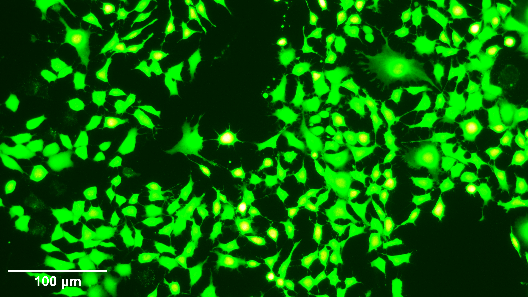

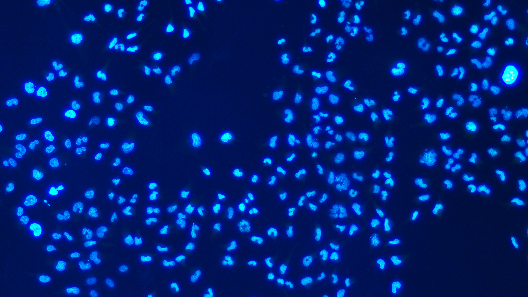


- 1 µM amorfrutin B


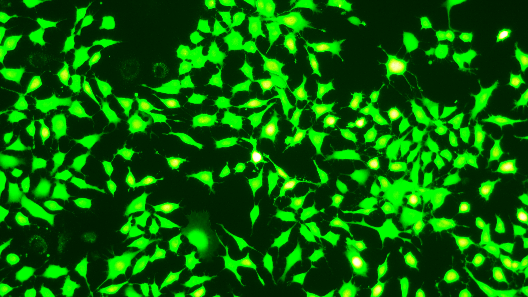

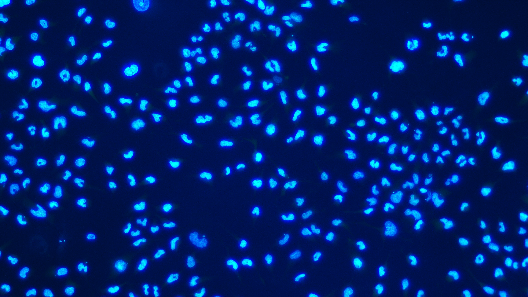


- 5 µM amorfrutin B


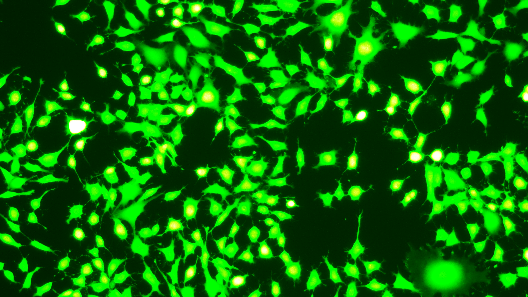

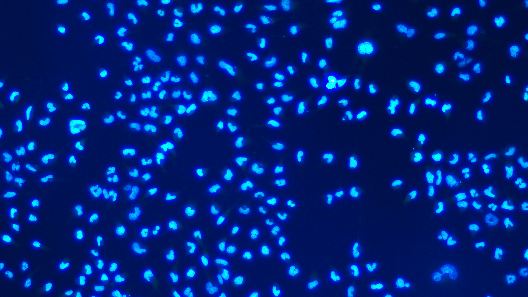


**Table S1.** Effects of amorfrutin B on studied parameters in microglia under normoxic conditions

a.

2+5 paradigm

| % of the control ± SEM | **Caspase-1 activity** |
| --- | --- |
| normoxia | 100 ± 3.20 |
| normoxia + amorfrutin B 1 µM | 87 ± 2.93 |
| normoxia + amorfrutin B 5 µM | 98 ± 3.47 |

6+18 paradigm

| % of the control ± SEM | **Caspase-1 activity** |
| --- | --- |
| normoxia | 100 ± 2.46 |
| normoxia + amorfrutin B 1 µM | 101 ± 3.92 |
| normoxia + amorfrutin B 5 µM | 90 ± 2.44 |

b.

| % of the control ± SEM | **Mitochondrial membrane potential** |
| --- | --- |
| normoxia | 100 ± 2.64 |
| normoxia + amorfrutin B 1 µM | 82 ± 2.23^***^ |
| normoxia + amorfrutin B 5 µM | 95 ± 2.56 |

c.

| % of the control ± SEM | **Metabolic activity** |
| --- | --- |
| normoxia | 100 ± 1.32 |
| normoxia + amorfrutin B 1 µM | 96 ± 2.43 |
| normoxia + amorfrutin B 5 µM | 106 ± 1.27 |

d.

| % of the control ± SEM | **LDH release** |
| --- | --- |
| normoxia | 100 ± 3.38 |
| normoxia + amorfrutin B 1 µM | 96 ± 2.07 |
| normoxia + amorfrutin B 5 µM | 88 ± 1.99^***^ |

e.

| % of the control ± SEM | **Proliferation potential** |
| --- | --- |
| normoxia | 100 ± 4.73 |
| normoxia + amorfrutin B 1 µM | 78 ± 4.65^**^ |
| normoxia + amorfrutin B 5 µM | 70 ± 3.56^***^ |

**Table S2.** Effects of amorfrutin B on studied parameters in neurons under normoxic conditions

a.

| % of the control ± SEM | **Viability** |
| --- | --- |
| normoxia | 100 ± 0.51 |
| normoxia + amorfrutin B 0.1 µM | 101 ± 0.46 |
| normoxia + amorfrutin B 1 µM | 102 ± 0.60 |
| normoxia + amorfrutin B 5 µM | 100 ± 0.58 |
| normoxia + amorfrutin B 10 µM | 95 ± 0.70 |

b.

| % of the control ± SEM | **Neurodegeneration level** |
| --- | --- |
| normoxia | 100 ± 0.75 |
| normoxia + amorfrutin B 1 µM | 103 ± 1.03 |
| normoxia + amorfrutin B 5 µM | 100 ± 1.23 |
| normoxia + amorfrutin B 1 µM  + GW9662 1 µM | 98 ± 1.27 |
| normoxia + amorfrutin B 5 µM  + GW9662 1 µM | 102 ± 1.22 |

DIFFERENCES IN THE EFFECTS OF THE HYPOXIC AND ISCHEMIC MODELS AND EFFECTS OF AMORFRUTIN B ACTION BETWEEN THE HYPOXIC AND ISCHEMIC CONDITIONS

**Figure S3.** There were significant differences in caspase-1 activity between hypoxic and ischemic cells treated with amorfrutin B in 1 µM (6+18 paradigm) and 5 µM concentration (6+18 and 2+5 paradigms), while no differences between vehicle treated hypoxic and ischemic cells were present


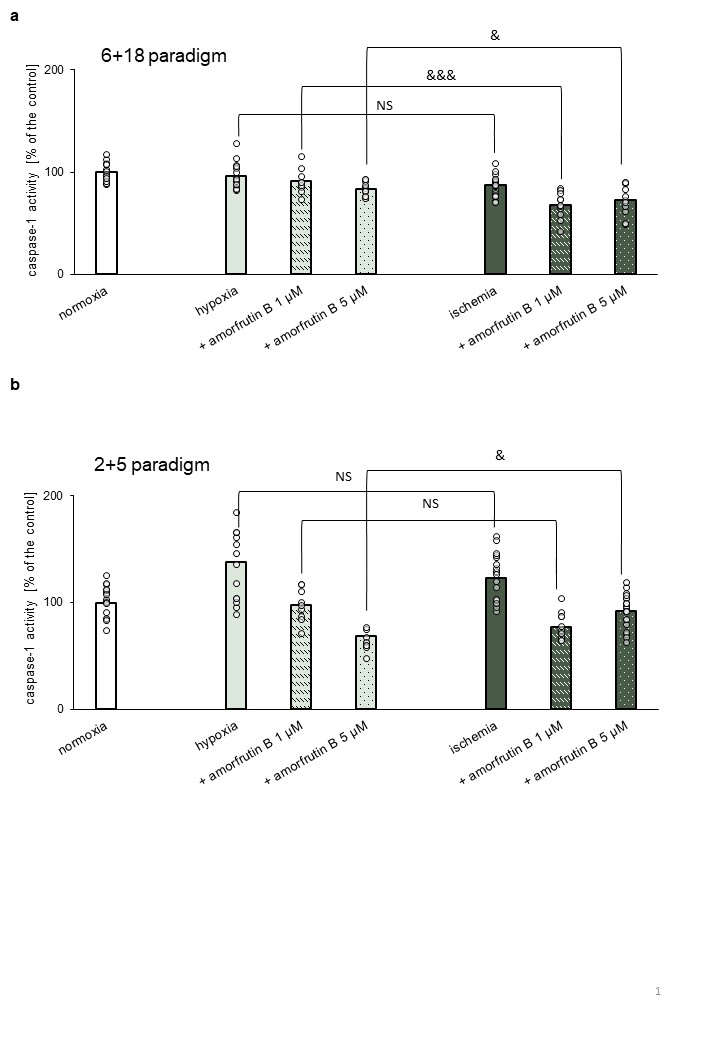


**Figure S4.** In the context of the expression of inflammation-related factors, the expression of IL-10 protein in hypoxia + amorfrutin B 1 µM vs ischemia + amorfrutin B 1 µM  differs


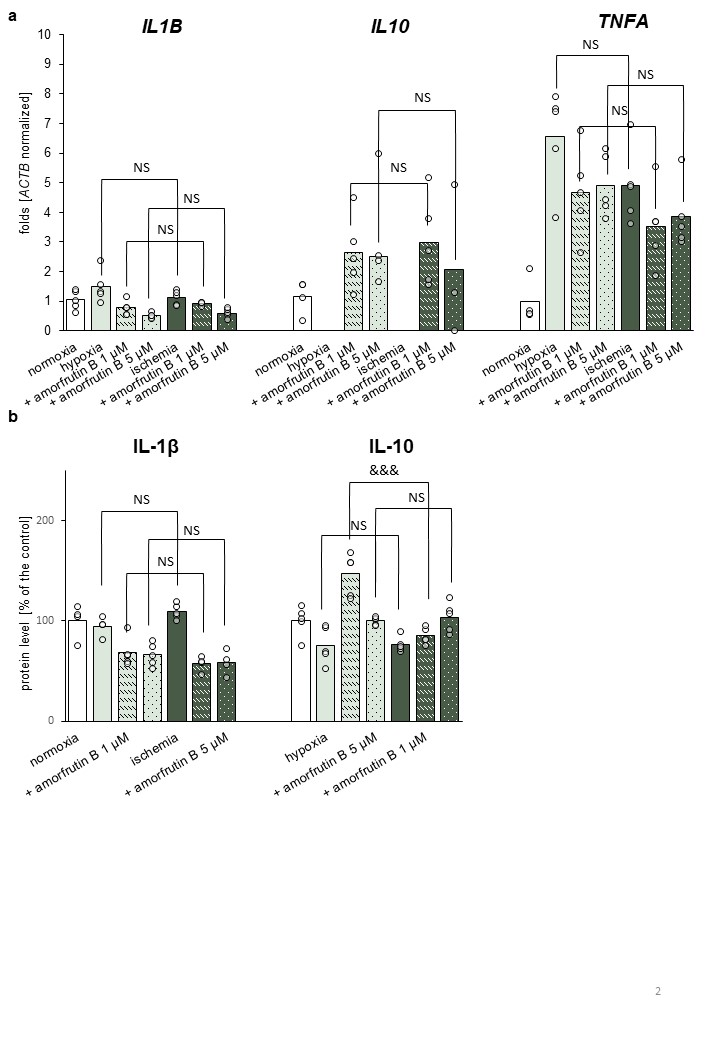


**Figure S5.** The expression of *PPARG*/PPARγ and *PGC1A*/PGC1α do not differ between hypoxic and ischemic groups treated with either vehicle or amorfrutin B


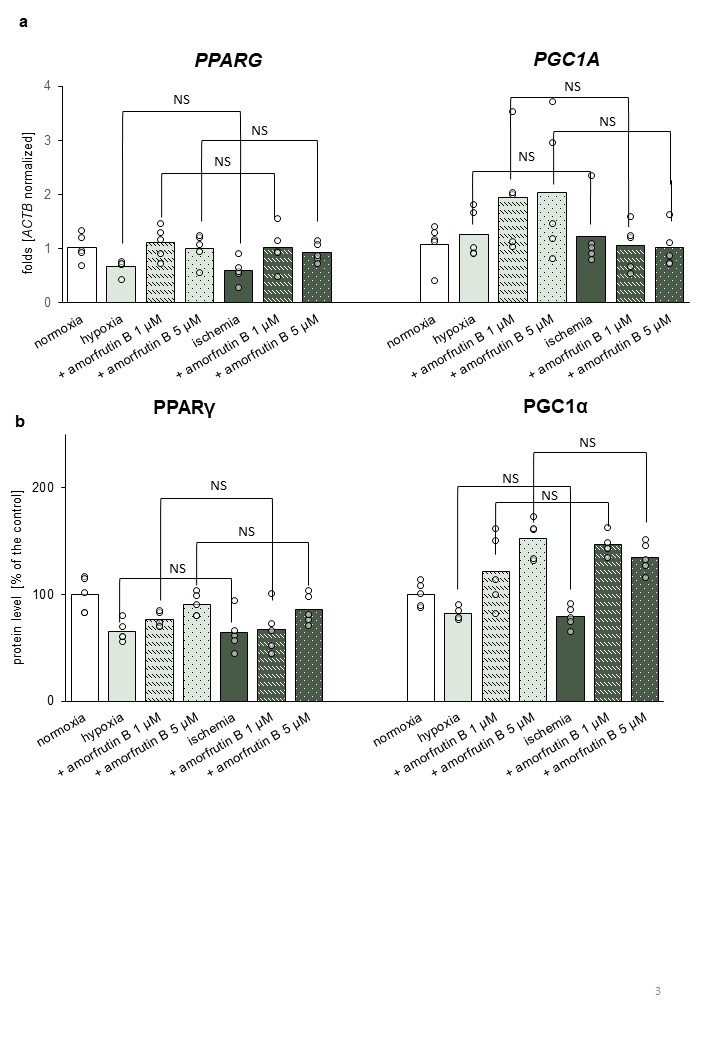


**Figure S6.** There is statistically significant difference in mitochondrial membrane potential between hypoxia + amorfrutin B 5 µM and ischemia + amorfrutin B 5 µM groups


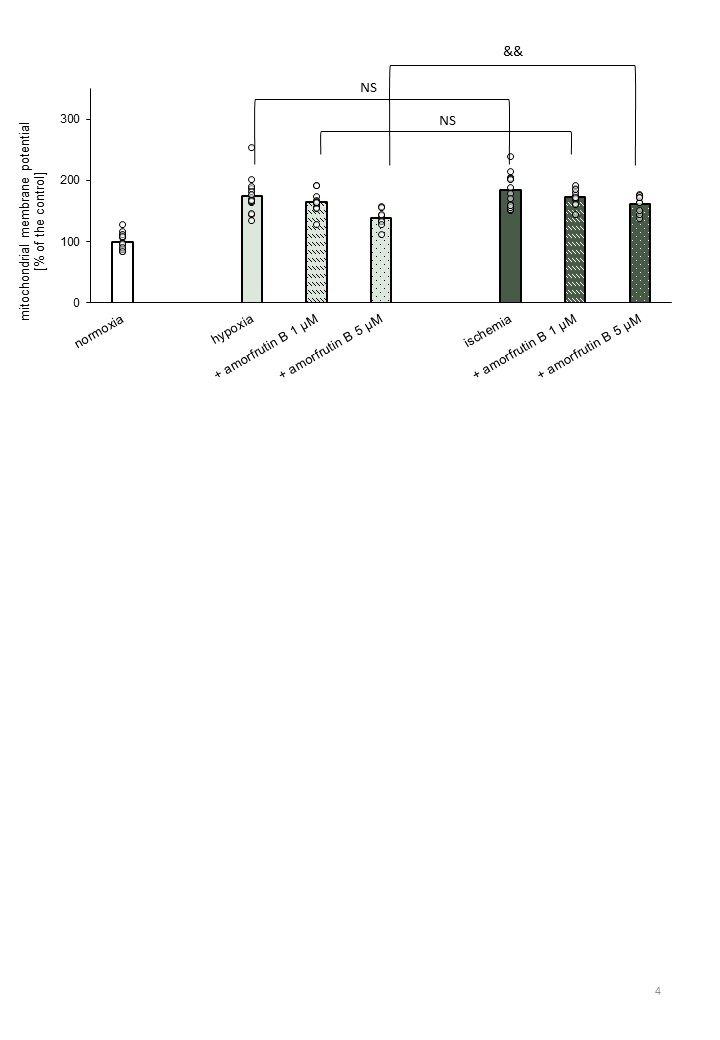


**Figure S7.** Cells subjected to hypoxia express higher level of BCL2 protein (b) than those subjected to ischemia

**
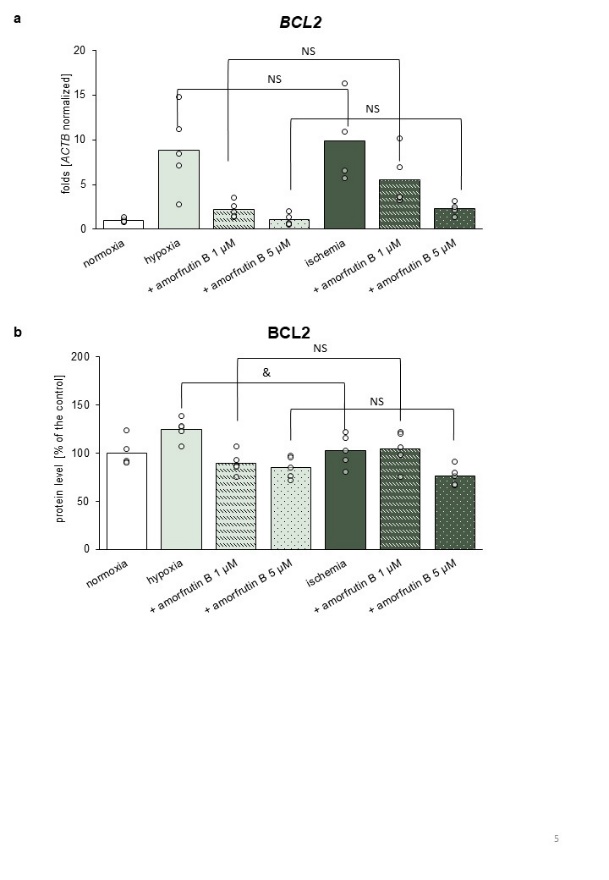
**

**Figure S8.** Hypoxic and ischemic groups (with or without amorfrutin B) do not differ in terms of ramification index (a). However, disparities are observed in cell body area (specifically between hypoxia + amorfrutin B 1 µM and ischemia + amorfrutin B 1 µM), minimum Feret diameter (between hypoxia + amorfrutin B 1 µM and ischemia + amorfrutin B 1 µM) and total number of cells (notably between hypoxia + amorfrutin B 5 µM and ischemia + amorfrutin B 5 µM) (a, b and c).


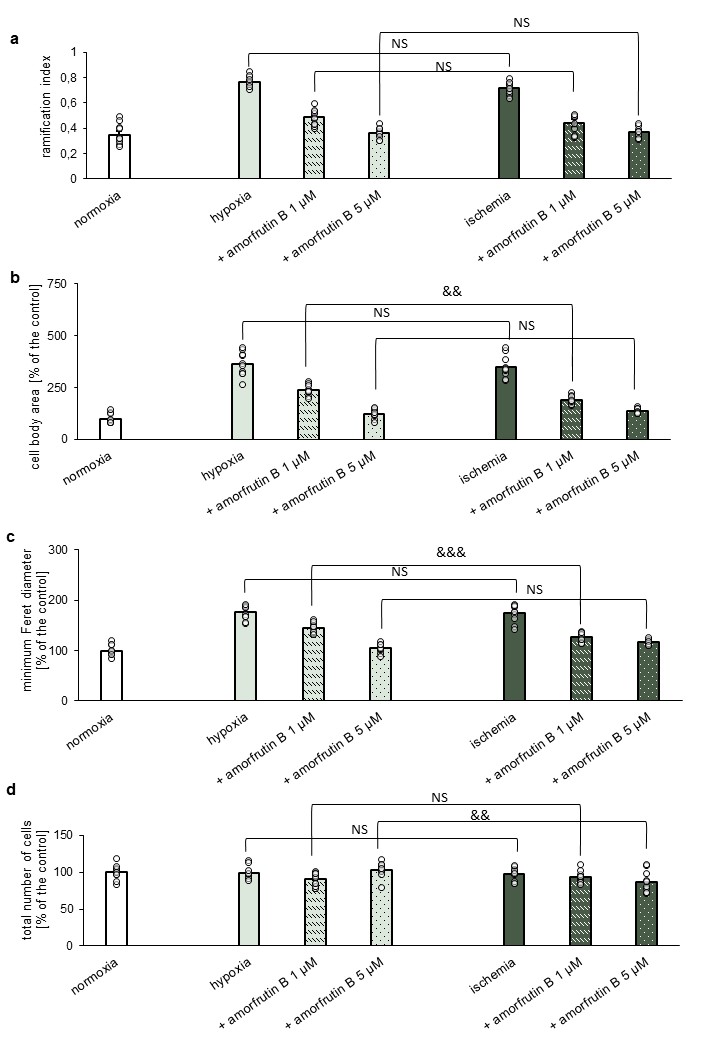


**Figure S9.** Hypoxic and ischemic groups (with or without amorfrutin B) do not differ in terms of MTT reduction and LDH release but differ in terms of  proliferation potential


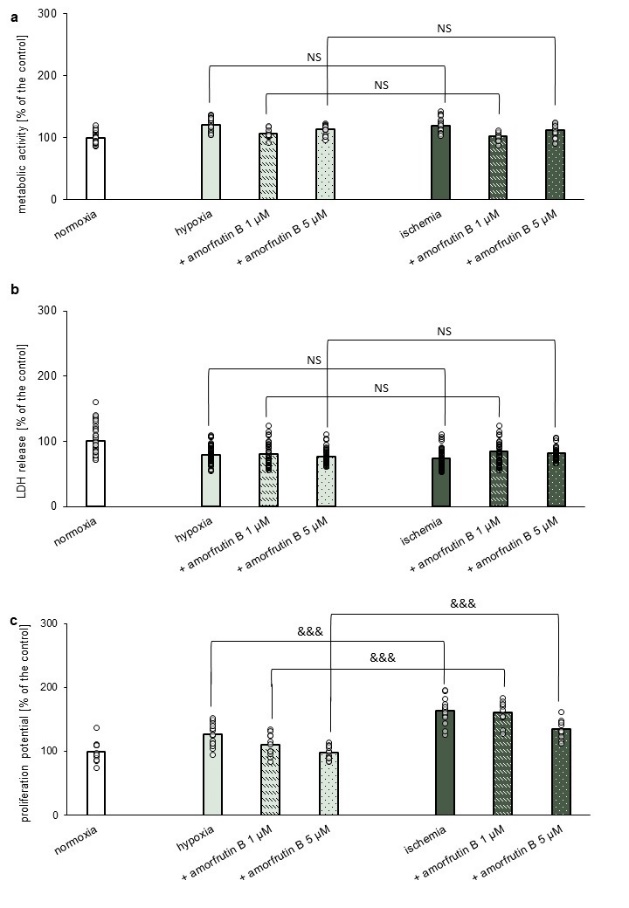


**Figure S10.** In neuronal cells, we observed significant differences in viability (a) between hypoxia- and ischemia-challenged cells, both with or without amorfrutin B (0.1 µM, 1 µM, 5 µM and 10 µM). In terms of the degree of neurodegeneration, we observed differences between hypoxia- and ischemia-subjected neurons treated with vehicle, amorfrutin B (1 and 5 µM) as well as amorfrutin B 1 µM + GW9662 1 µM (b)

**
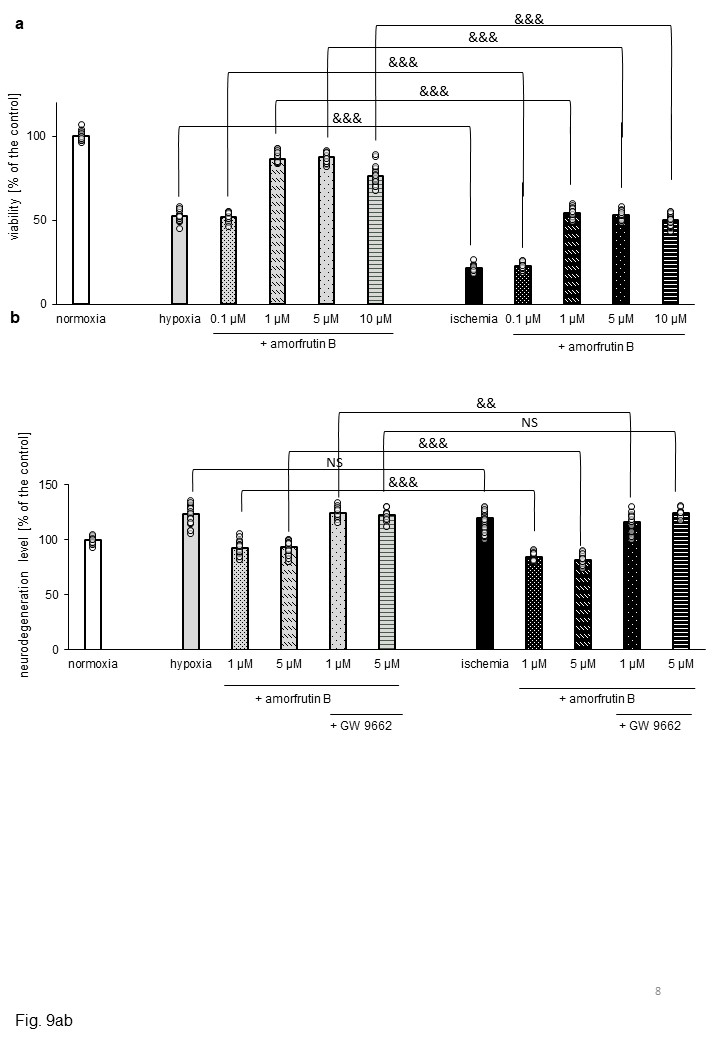
**
